# Collaborative orchestration of BH3-only proteins governs Bak/Bax-dependent hepatocyte apoptosis under antiapoptotic protein-deficiency in mice

**DOI:** 10.1038/s41418-025-01458-y

**Published:** 2025-02-24

**Authors:** Shinnosuke Kudo, Hayato Hikita, Yoshinobu Saito, Kazuhiro Murai, Takahiro Kodama, Tomohide Tatsumi, Tetsuo Takehara

**Affiliations:** Department of Gastroenterology and Hepatology, Osaka University Graduate School of Medicine, Osaka, Japan

**Keywords:** Cell biology, Proteins, Physiology

## Abstract

The fine-tuned balance between anti-apoptotic Bcl-2 family proteins, such as Bcl-xL and Mcl-1, and pro-apoptotic Bcl-2 family proteins, like Bak and Bax, is crucial for maintaining hepatocyte integrity. BH3-only proteins, including Bid, Bim, Puma, Noxa, Bad, Bmf, Bik and Hrk, serve as apoptosis initiators. They are activated by various stimuli, which leads to Bak/Bax activation. We previously reported that Bid and Bim contributed to hepatocyte apoptosis through Bak/Bax activation in the absence of anti-apoptotic proteins Bcl-xL and/or Mcl-1. However, the comprehensive involvement of all eight BH3-only proteins in Bak/Bax-dependent hepatocyte apoptosis remains unclear. Puma disruption suppressed hepatocyte apoptosis in hepatocyte-specific Bcl-xL or Mcl-1 knockout (Bcl-xL^ΔHep/ΔHep^ or Mcl-1^ΔHep/ΔHep^) mice. Disruption of Bid and Bim partially prevented lethality in Mcl-1^ΔHep/+^ Bcl-xL^ΔHep/ΔHep^ mice, although severe hepatocyte apoptosis persisted, which was suppressed by additional Puma disruption. However, hepatocyte apoptosis was still induced compared to that in Mcl-1^ΔHep/+^ Bcl-xL^ΔHep/ΔHep^ Bax^ΔHep/ΔHep^ Bak^−/−^ mice. Triple disruption of Bid, Bim and Puma did not prevent induction of hepatocyte apoptosis in tamoxifen-induced Mcl-1^iΔHep/iΔHep^ Bcl-xL^iΔHep/iΔHep^ mice. Primary hepatocytes, isolated from Mcl-1^fl/fl^ Bcl-xL^fl/fl^ Bid^−/−^ Bim^−/−^ Puma^−/−^ mice and immortalized, underwent apoptosis with doxycycline-dependent Cre recombination. Among the remaining five BH3-only proteins, Bik and Hrk were not expressed in these cells, and Noxa knockdown, but not Bad or Bmf knockdown, reduced apoptosis. Noxa disruption alleviated hepatocyte apoptosis in Mcl-1^ΔHep/ΔHep^ mice and tamoxifen-induced Mcl-1^iΔHep/iΔHep^ Bcl-xL^iΔHep/iΔHep^ Bid^−/−^ Bim^−/−^ Puma^−/−^ mice, prolonging survival. Apoptosis persisted in immortalized primary hepatocytes isolated from Mcl-1^fl/fl^ Bcl-xL^fl/fl^ Bid^−/−^ Bim^−/−^ Puma^−/−^ Noxa^−/−^ mice where doxycycline-dependent Cre recombination was induced, but was completely suppressed by Bak/Bax knockdown, while Bad or Bmf knockdown had no effect. In conclusion, among the eight BH3-only proteins, Puma and Noxa, alongside Bid and Bim, contributed to Bak/Bax-dependent hepatocyte apoptosis, but not indispensably, in the absence of Mcl-1 and Bcl-xL.

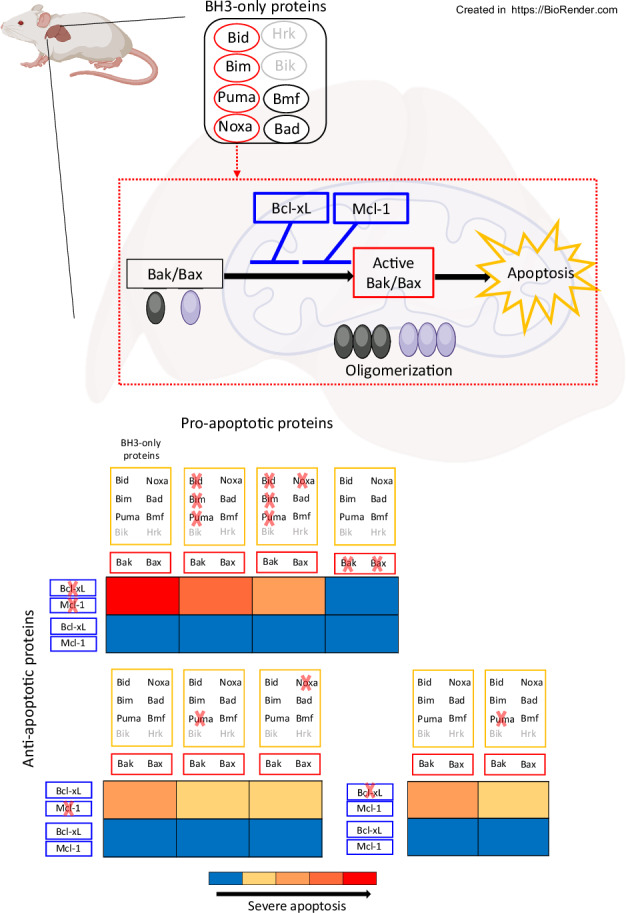

## Introduction

The mitochondrial pathway of hepatocyte apoptosis is orchestrated by members of the B-cell lymphoma-2 (Bcl-2) family proteins, which include the anti-apoptotic proteins Bcl-2, B-cell lymphoma-extra large (Bcl-xL), myeloid cell leukemia-1 (Mcl-1), B-cell lymphoma-w (Bcl-w), and Bcl-2-related protein A1 (Bcl2A1), as well as the pro-apoptotic proteins Bcl-2-antagonist/killer (Bak) and Bcl-2-associated X protein (Bax) [1]. Pro-apoptotic proteins act as effector molecules in this apoptotic cascade [2]. Upon activation, they form pores in the mitochondrial outer membrane, facilitating the release of cytochrome c. This leads to the activation of the caspase cascade and finally leads to apoptosis [3]. Anti-apoptotic Bcl-2 family members, including Bcl-xL and Mcl-1, inhibit the mitochondrial pathway of apoptosis by neutralizing Bak/Bax activity [4]. Bcl-2 homology domain 3 (BH3)-only proteins, a group of eight members, Bid, Bim, Puma, Noxa, Bad, Bmf, Bik and Hrk, are pro-apoptotic proteins [5], and the mechanism by which these proteins activate Bak/Bax has long been debated, centering on the “direct activation model” versus the “indirect activation model” [6, 7].

The direct activation model proposes that BH3-only proteins can be divided into two groups: activators and sensitizers [8–10]. Activator BH3-only proteins directly activate Bak/Bax to induce apoptosis, whereas sensitizer BH3-only proteins cannot directly activate Bak/Bax but bind to anti-apoptotic Bcl-2 family members, allowing sequestered activators to activate Bak/Bax. Among the eight BH3-only proteins, Bid, Bim and Puma are generally classified as activators [11, 12], whereas others, such as Bad, are classified as sensitizers [8, 13]. Although the direct activation model has been broadly accepted for decades, several studies have challenged it, leading to the proposal of the indirect activation model.

The indirect model proposes that BH3-only proteins cannot activate Bak/Bax directly and that all BH3-only proteins are sensitizers. They bind to anti-apoptotic Bcl-2 family members Bcl-xL and Mcl-1, neutralizing them and thereby enabling Bak/Bax activation to induce apoptosis [14–16]. Recent in vitro studies using CRISPR-Cas9 technology have further revealed that, in the indirect model, the mitochondrial outer membrane acts as an activator to initiate Bak/Bax oligomerization, providing strong evidence of this indirect activation model [17, 18]. Although the role of BH3-only proteins was precisely analyzed by using in vitro models, how Bak/Bax are activated by BH3-only proteins in vivo, including in hepatocytes, is not yet fully understood.

We previously reported that hepatocyte-specific Mcl-1 knockout (KO) (*Mcl-1*^*flox/flox*^ *Alb-Cre*) mice (Mcl-1^ΔHep/ΔHep^ mice) and hepatocyte-specific Bcl-xL KO (*Bcl-xL*^*flox/flox*^ *Alb-Cre*) mice (Bcl-xL^ΔHep/ΔHep^ mice) exhibit spontaneous hepatocyte apoptosis [19, 20]. Additionally, Bak or Bax knockout suppressed hepatocyte apoptosis in these mice [19, 21], demonstrating that apoptosis caused by the deletion of Mcl-1 and/or Bcl-xL is dependent on Bak and Bax. Using these Bak/Bax-dependent apoptosis model mice, we previously examined the roles of BH3-only proteins, Bid and Bim, and reported that the disruption of Bid and Bim decreased hepatocyte apoptosis in Bcl-xL^ΔHep/ΔHep^ mice and Mcl-1^ΔHep/ΔHep^ mice [22], suggesting that among the eight BH3-only proteins, Bid and Bim are important for Bak/Bax-dependent apoptosis in hepatocytes. However, the involvement of other BH3-only proteins has yet to be elucidated. In the present study, we aimed to investigate the comprehensive involvement of BH3-only proteins in Bak/Bax-dependent hepatocyte apoptosis in the absence of anti-apoptotic Bcl-2 family proteins.

In the present study, we demonstrate for the first time, that among the eight BH3-only proteins, Puma and Noxa, along with Bid and Bim, are involved in the execution of Bak/Bax-dependent hepatocyte apoptosis caused by the absence of anti-apoptotic Bcl-2 family proteins. Our study revealed the orchestrated role of BH3-only proteins in the murine liver.

## Results

### Disruption of Puma suppresses hepatocyte apoptosis in mice with hepatocyte-specific knockout of Bcl-xL or Mcl-1

**Fig. 1:**
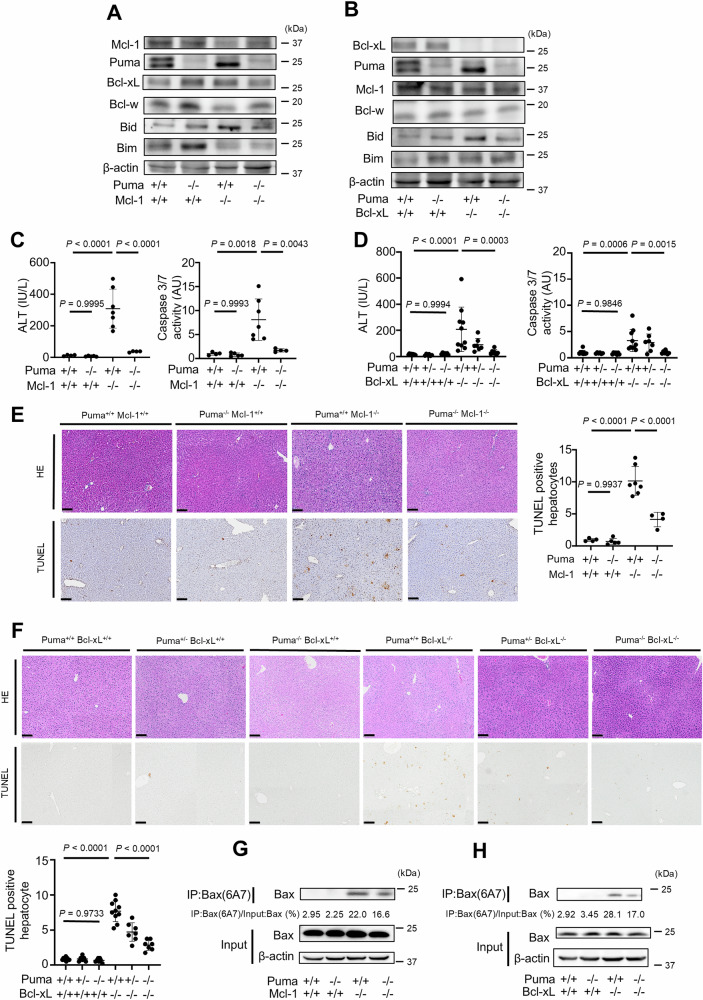
Disruption of Puma suppresses hepatocyte apoptosis in mice with hepatocyte-specific knockout of Bcl-xL or Mcl-1. We generated *Bcl-xL*^*flox/flox*^ *Alb-Cre Puma*^*−/−*^ mice, *Mcl-1*^*flox/flox*^ *Alb-Cre Puma*^*−/−*^ mice and control mice. These mice were analyzed at 6–8 weeks of age; *n* ≥ 4 per group. **A**, **B** Western blotting analysis of liver lysates. **C**, **D** Serum ALT levels and caspase 3/7 activity. **E**, **F** Representative images of H&E staining and TUNEL staining and their quantitative results. **G**, **H** The active form of Bax was detected by immunoprecipitation for Bax 6A7. The ratio of Bax in the post-immunoprecipitation samples to that in the whole cell extract samples was calculated. Data is represented as mean ± SD. Data was analyzed by One-way ANOVA with Sidak’s multiple comparisons tests (**C–F**). Scale bar (**E**, **F**): 100 μm.

We initially examined the role of another BH3-only protein, Puma, in Bak/Bax activation in the absence of Bcl-xL or Mcl-1. We crossed Mcl-1^ΔHep/ΔHep^ mice or Bcl-xL^ΔHep/ΔHep^ mice with Puma KO (*Puma*^*−/−*^) mice (Puma^−/−^ mice) and generated Mcl-1^ΔHep/Δhep^ Puma^−/−^ mice and Bcl-xL^Δhep/Δhep^ Puma^−/−^ mice. The expression levels of Mcl-1 and Puma were reduced in the liver tissue of Mcl-1^ΔHep/ΔHep^ Puma^−/−^ mice (Fig. 1A). Similarly, Bcl-xL and Puma expression in the livers of Bcl-xL^ΔHep/ΔHep^ Puma^−/−^ mice was reduced (Fig. 1B). Bcl-w was detected in the whole-liver lysates of these mice (Fig. 1A, B), whereas expression levels of Bcl-2 and Bcl-2A1 were nearly undetectable (Supplementary Fig. 1A). Bcl-2 and Bcl-2A1 were also barely detectable in mouse primary hepatocytes (Supplementary Fig. 1A). Mcl-1^ΔHep/ΔHep^ Puma^−/−^ mice and Bcl-xL^ΔHep/ΔHep^ Puma^−/−^ mice displayed significantly lower serum alanine transaminase (ALT) levels and reduced serum caspase 3/7 activity than Mcl-1^ΔHep/ΔHep^ mice and Bcl-xL^ΔHep/ΔHep^ mice, respectively (Fig. 1C, D). These mice also presented a smaller number of terminal deoxynucleotidyl transferase-mediated deoxyuridine triphosphate nick-end labeling (TUNEL)-positive hepatocytes (Fig. 1E, F). We performed Western blotting and immunoprecipitation using the Bax antibody clone 6A7 (Bax(6A7)), which selectively recognizes the conformationally active form of Bax [23–25]. Monoclonal 6A7 antibody recognizes Bax only after helix 1 has been unfolded and 6A7 epitope was exposed [26, 27]. This antibody seems to detect both homodimerized Bax and heterodimerization-free state of Bax [27]. Western blotting and immunoprecipitation of Bax 6a7 revealed that livers from Mcl-1^ΔHep/ΔHep^ Puma^−/−^ mice and Bcl-xL^ΔHep/ΔHep^ Puma^−/−^ mice presented lower expression levels of active Bax than those from Mcl-1^ΔHep/ΔHep^ mice and Bcl-xL^ΔHep/ΔHep^ mice did (Fig. 1G, H). These results demonstrated that the BH3-only protein Puma was involved in executing hepatocyte apoptosis via Bak/Bax activation in the absence of anti-apoptotic Bcl-2 family proteins.

### Disruption of Bid, Bim and Puma cannot fully suppress hepatocyte apoptosis induced by the deletion of Bcl-xL and Mcl-1

**Fig. 2:**
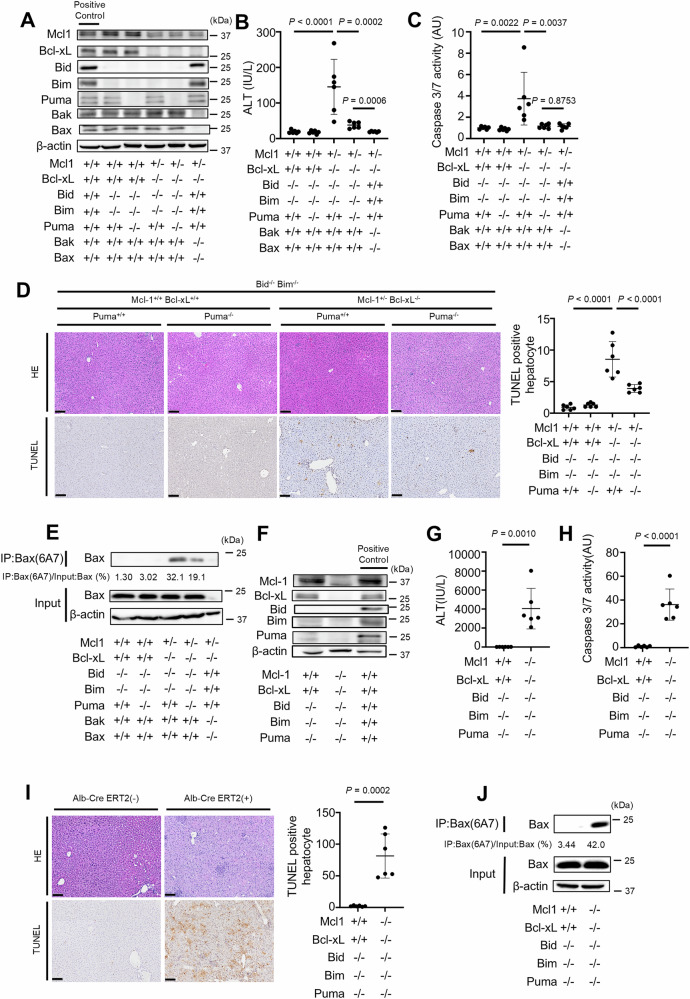
Disruption of Bid, Bim and Puma cannot fully suppress hepatocyte apoptosis induced by the deletion of Bcl-xL and Mcl-1. **A**–**E** We generated *Bcl-xL*^*flox/flox*^ *Mcl-1*^*flox/+*^ *Alb-Cre Bid*^*−/−*^ *Bim*^*−/−*^ *Puma*^*+/+*^ mice, *Bcl-xL*^*flox/flox*^ *Mcl-1*^*flox/+*^ *Alb-Cre Bid*^*−/−*^ *Bim*^*−/−*^ *Puma*^*−/−*^ mice, and control mice. We also generated *Bcl-xL*^*flox/flox*^ *Mcl-1*^*flox/+*^ *Bax*^*fl/fl*^ *Alb-Cre Bak*^−/−^ mice. These mice were analyzed at 6–8 weeks of age; n = 6 per group. **A** Western blotting analysis of liver lysates. C57BL/6J mouse was used as a positive control. **B**, **C** Serum ALT levels and Caspase 3/7 activity. **D** Representative images of H&E staining and TUNEL staining and their quantitative results. **E** Immunoprecipitation of Bax6A7. The ratio of Bax in the post-immunoprecipitation samples to that in the whole cell extract samples was calculated. **F**–**J** We generated *Bcl-xL*^*flox/flox*^ *Mcl-1*^*flox/flox*^ *Alb-Cre*^*ERT2*^ *Bid*^*−/−*^ *Bim*^*−/−*^ *Puma*^*−/−*^ mice and control mice. These mice were injected intraperitoneally with 1 mg of tamoxifen for 3 consecutive days and analyzed 24 h after the last tamoxifen injection. **F** Western blotting analysis of liver lysates after tamoxifen injection. C57BL/6J mouse was used as a positive control. **G**, **H** Serum ALT levels and caspase 3/7 activity. **I** Representative images of H&E staining and TUNEL staining and their quantitative results. **J** Immunoprecipitation of Bax6A7 and the ratio of Bax in the post-immunoprecipitation samples to that in the whole cell extract samples. Data is represented as mean ± SD. Data was analyzed by One-way ANOVA with Sidak’s multiple comparisons tests (**B**–**D**) or two-tailed unpaired *t* tests (**G**–**I**). Scale bar (**D**, **I**): 100 μm.

We next examined whether disruption of Bid, Bim and Puma could block hepatocyte Bak/Bax-dependent apoptosis in the absence of Bcl-xL and Mcl-1. We have previously demonstrated that Bcl-xL^ΔHep/ΔHep^ Mcl-1^ΔHep/ΔHep^ mice, as well as Bcl-xL^ΔHep/ΔHep^ Mcl-1^ΔHep/+^ mice, exhibit impaired liver development and that all Bcl-xL^ΔHep/ΔHep^ Mcl-1^ΔHep/ΔHep^ mice die within one day after birth [28]. Disruption of Bid, Bim and Puma could not reduce the mortality of Bcl-xL^ΔHep/ΔHep^ Mcl-1^ΔHep/ ΔHep^ mice (Tables 1 and 2). Bcl-xL^ΔHep/ΔHep^ Mcl-1^ΔHep/+^ mice also exhibited severe hepatocyte apoptosis, but few Bcl-xL^ΔHep/ΔHep^ Mcl-1^ΔHep/+^ mice survived. A surviving mouse showed severe elevation of serum ALT (ALT = 1357 IU/L, *n* = 1) and massive TUNEL-positive hepatocytes (74 positive cells/field, *n* = 1, Supplementary Fig. 2A) aged 6 weeks. Disruption of both Bid and Bim reduced the serum ALT levels and the number of TUNEL-positive hepatocytes compared with those in a surviving Bcl-xL^ΔHep/ΔHep^ Mcl-1^ΔHep/+^ mouse (Fig. 2B, D), but Bcl-xL^ΔHep/ΔHep^ Mcl-1^ΔHep/+^ Bid^−/−^ Bim^−/−^ mice still exhibited hepatocyte apoptosis (Fig. 2A–D). Additional disruption of Puma significantly reduced the serum ALT level, serum caspase 3/7 activity, number of TUNEL-positive hepatocytes and expression of active Bax in Bcl-xL^ΔHep/ΔHep^ Mcl-1^ΔHep/+^ Bid^−/−^ Bim^−/−^ mice (Fig. 2B–E). However, in Bcl-xL^ΔHep/ΔHep^ Mcl-1^ΔHep/+^ Bid^−/−^ Bim^−/−^ Puma^−/−^ mice, the serum ALT levels and the expression of active Bax in their liver tissues remained higher than those in Bcl-xL^ΔHep/ΔHep^ Mcl-1^ΔHep/+^ Bax^ΔHep/ΔHep^ Bak^−/−^ mice (Fig. 2B, E).

**Table 1:** Genotypes of offspring from the mating of *Bcl-xL*^*flox/flox*^ *Mcl-1*^*flox/+*^ *Alb-Cre Bid*^*−/−*^ *Bim*^*−/−*^ (or *Bim*^*+/−*^) mice and *Bcl-xL*^*flox/flox*^ *Mcl-1*^*flox/flox*^ *Bid*^*−/−*^ *Bim*^*−/−*^ (or *Bim*^*+/−*^) mice were analyzed at embryonic 18.5 day and 3-4 weeks of age.

Age		Genotype		Born ratio	
Embryonic 18.5 day		*Bcl-xL*^*flox/flox*^ *Mcl-1*^*flox/+*^ *Bid*^*−/−*^ *Bim*^*+/−*^	*Bcl-xL*^*flox/flox*^ *Mcl-1*^*flox/+*^ *Alb-Cre(+) Bid*^*−/−*^ *Bim*^*+/−*^	5.9% (6/101)	4.0% (4/101)
		(Bcl-xL^+/+^ Mcl-1^+/+^ Bid^−/−^ Bim^+/−^)	(Bcl-xL^ΔHep/ΔHep^ Mcl-1^ΔHep/+^ Bid^−/−^ Bim^+/−^)		
		*Bcl-xL*^*flox/flox*^ *Mcl-1*^*flox/+*^ *Bid*^*−/−*^ *Bim*^*−/−*^	*Bcl-xL*^*flox/flox*^ *Mcl-1*^*flox/+*^ *Alb-Cre(+) Bid*^*−/−*^ *Bim*^*−/−*^	21.8% (22/101)	17.8% (18/101)
		(Bcl-xL^+/+^ Mcl-1^+/+^ Bid^−/−^ Bim^−/−^)	(Bcl-xL^ΔHep/ΔHep^ Mcl-1^ΔHep/+^ Bid^−/−^ Bim^−/−^)		
		*Bcl-xL*^*flox/flox*^ *Mcl-1*^*flox/flox*^ *Bid*^*−/−*^ *Bim*^*+/−*^	*Bcl-xL*^*flox/flox*^ *Mcl-1*^*flox/flox*^ *Alb-Cre(+) Bid*^*−/−*^ *Bim*^*+/−*^	5.0% (5/101)	11.9% (12/101)
		(Bcl-xL^+/+^ Mcl-1^+/+^ Bid^−/−^ Bim^+/−^)	(Bcl-xL^ΔHep/ΔHep^ Mcl-1^ΔHep/ΔHep^ Bid^−/−^ Bim^+/−^)		
		*Bcl-xL*^*flox/flox*^ *Mcl-1*^*flox/flox*^ *Bid*^*−/−*^ *Bim*^*−/−*^	*Bcl-xL*^*flox/flox*^ *Mcl-1*^*flox/flox*^ *Alb-Cre(+) Bid*^*−/−*^ *Bim*^*−/−*^	15.8% (16/101)	17.8% (18/101)
		(Bcl-xL^+/+^ Mcl-1^+/+^ Bid^−/−^ Bim^−/−^)	(Bcl-xL^ΔHep/ΔHep^ Mcl-1^ΔHep/ΔHep^ Bid^−/−^ Bim^−/−^)		
3-4 weeks of age		*Bcl-xL*^*flox/flox*^ *Mcl-1*^*flox/+*^ *Bid*^*−/−*^ *Bim*^*+/−*^	*Bcl-xL*^*flox/flox*^ *Mcl-1*^*flox/+*^ *Alb-Cre(+) Bid*^*−/−*^ *Bim*^*+/−*^	23.4% (111/474)	8.9% (42/474)
		(Bcl-xL^+/+^ Mcl-1^+/+^ Bid^−/−^ Bim^+/−^)	(Bcl-xL^ΔHep/ΔHep^ Mcl-1^ΔHep/+^ Bid^−/−^ Bim^+/−^)		
		*Bcl-xL*^*flox/flox*^ *Mcl-1*^*flox/+*^ *Bid*^*−/−*^ *Bim*^*−/−*^	*Bcl-xL*^*flox/flox*^ *Mcl-1*^*flox/+*^ *Alb-Cre(+) Bid*^*−/−*^ *Bim*^*−/−*^	15.6% (74/474)	12.2% (58/474)
		(Bcl-xL^+/+^ Mcl-1^+/+^ Bid^−/−^ Bim^−/−^)	(Bcl-xL^ΔHep/ΔHep^ Mcl-1^ΔHep/+^ Bid^−/−^ Bim^−/−^)		
		*Bcl-xL*^*flox/flox*^ *Mcl-1*^*flox/flox*^ *Bid*^*−/−*^ *Bim*^*+/−*^	*Bcl-xL*^*flox/flox*^ *Mcl-1*^*flox/flox*^ *Alb-Cre(+) Bid*^*−/−*^ *Bim*^*+/−*^	25.7% (122/474)	0% (0/474)
		(Bcl-xL^+/+^ Mcl-1^+/+^ Bid^−/−^ Bim^+/−^)	(Bcl-xL^ΔHep/ΔHep^ Mcl-1^ΔHep/ΔHep^ Bid^−/−^ Bim^+/−^)		
		*Bcl-xL*^*flox/flox*^ *Mcl-1*^*flox/flox*^ *Bid*^*−/−*^ *Bim*^*−/−*^	*Bcl-xL*^*flox/flox*^ *Mcl-1*^*flox/flox*^ *Alb-Cre(+) Bid*^*−/−*^ *Bim*^*−/−*^	14.1% (67/474)	0% (0/474)
		(Bcl-xL^+/+^ Mcl-1^+/+^ Bid^−/−^ Bim^−/−^)	(Bcl-xL^ΔHep/ΔHep^ Mcl-1^ΔHep/ΔHep^ Bid^−/−^ Bim^−/−^)		

Each *Alb-Cre(−)* and *Alb-Cre(+)* was expected to be born a 1:1 ratio.

**Table 2:** Genotypes of offspring from the mating of Bcl-xL^flox/flox^ Mcl-1^flox/+^ Alb-Cre Bid^−/−^ Bim^−/−^ (or Bim^+/−^) Puma^+/−^ mice and Bcl-xL^flox/flox^ Mcl-1^flox/flox^ Bid^−/−^ Bim^−/−^ (or Bim^+/−^) Puma^+/−^ mice were analyzed at 3-4 weeks of age.

Age		Genotype		Born ratio	
3-4 weeks of age		*Bcl-xL*^*flox/flox*^ *Mcl-1*^*flox/+*^ *Bid*^*−/−*^ *Bim*^*+/−*^ *Puma*^*+/+*^	*Bcl-xL*^*flox/flox*^ *Mcl-1*^*flox/+*^ *Alb-Cre(+) Bid*^*−/−*^ *Bim*^*+/−*^ *Puma*^*+/+*^	4.1% (9/217)	1.8% (4/217)
		(Bcl-xL^+/+^ Mcl-1^+/+^ Bid^−/−^ Bim^+/−^ Puma^+/+^)	(Bcl-xL^ΔHep/ΔHep^ Mcl-1^ΔHep/+^ Bid^−/−^ Bim^+/−^ Puma^+/+^)		
		*Bcl-xL*^*flox/flox*^ *Mcl-1*^*flox/+*^ *Bid*^*−/−*^ *Bim*^*+/−*^ *Puma*^*+/−*^	*Bcl-xL*^*flox/flox*^ *Mcl-1*^*flox/+*^ *Alb-Cre(+) Bid*^*−/−*^ *Bim*^*+/−*^ *Puma*^*+/−*^	9.2% (20/217)	10.1% (22/217)
		(Bcl-xL^+/+^ Mcl-1^+/+^ Bid^−/−^ Bim^+/−^ Puma^+/−^)	(Bcl-xL^ΔHep/ΔHep^ Mcl-1^ΔHep/+^ Bid^−/−^ Bim^+/−^ Puma^+/−^)		
		*Bcl-xL*^*flox/flox*^ *Mcl-1*^*flox/+*^ *Bid*^*−/−*^ *Bim*^*+/−*^ *Puma*^*−/−*^	*Bcl-xL*^*flox/flox*^ *Mcl-1*^*flox/+*^ *Alb-Cre(+) Bid*^*−/−*^ *Bim*^*+/−*^ *Puma*^*−/−*^	5.5% (12/217)	3.2% (7/217)
		(Bcl-xL^+/+^ Mcl-1^+/+^ Bid^−/−^ Bim^+/−^ Puma^−/−^)	(Bcl-xL^ΔHep/ΔHep^ Mcl-1^ΔHep/+^ Bid^−/−^ Bim^+/−^ Puma^−/−^)		
		*Bcl-xL*^*flox/flox*^ *Mcl-1*^*flox/+*^ *Bid*^*−/−*^ *Bim*^*−/−*^ *Puma*^*+/+*^	*Bcl-xL*^*flox/flox*^ *Mcl-1*^*flox/+*^ *Alb-Cre(+) Bid*^*−/−*^ *Bim*^*−/−*^ *Puma*^*+/+*^	4.1% (9/217)	3.2% (7/217)
		(Bcl-xL^+/+^ Mcl-1^+/+^ Bid^−/−^ Bim^−/−^ Puma^+/+^)	(Bcl-xL^ΔHep/ΔHep^ Mcl-1^ΔHep/+^ Bid^−/−^ Bim^−/−^ Puma^+/+^)		
		*Bcl-xL*^*flox/flox*^ *Mcl-1*^*flox/+*^ *Bid*^*−/−*^ *Bim*^*−/−*^ *Puma*^*+/−*^	*Bcl-xL*^*flox/flox*^ *Mcl-1*^*flox/+*^ *Alb-Cre(+) Bid*^*−/−*^ *Bim*^*−/−*^ *Puma*^*+/−*^	12.0% (26/217)	9.2% (20/217)
		(Bcl-xL^+/+^ Mcl-1^+/+^ Bid^−/−^ Bim^−/−^ Puma^+/−^)	(Bcl-xL^ΔHep/ΔHep^ Mcl-1^ΔHep/+^ Bid^−/−^ Bim^−/−^ Puma^+/−^)		
		*Bcl-xL*^*flox/flox*^ *Mcl-1*^*flox/+*^ *Bid*^*−/−*^ *Bim*^*−/−*^ *Puma*^*−/−*^	*Bcl-xL*^*flox/flox*^ *Mcl-1*^*flox/+*^ *Alb-Cre(+) Bid*^*−/−*^ *Bim*^*−/−*^ *Puma*^*−/−*^	5.5% (12/217)	10.1% (22/217)
		(Bcl-xL^+/+^ Mcl-1^+/+^ Bid^−/−^ Bim^−/−^ Puma^−/−^	(Bcl-xL^ΔHep/ΔHep^ Mcl-1^ΔHep/+^ Bid^−/−^ Bim^−/−^ Puma^−/−^)		
		*Bcl-xL*^*flox/flox*^ *Mcl-1*^*flox/flox*^ *Bid*^*−/−*^ *Bim*^*+/−*^ *Puma*^*+/+*^	*Bcl-xL*^*flox/flox*^ *Mcl-1*^*flox/flox*^ *Alb-Cre(+) Bid*^*−/−*^ *Bim*^*+/−*^ *Puma*^*+/+*^	0.9% (2/217)	0% (0/217)
		(Bcl-xL^+/+^ Mcl-1^+/+^ Bid^−/−^ Bim^+/−^ Puma^+/+^)	(Bcl-xL^ΔHep/ΔHep^ Mcl-1^ΔHep/ΔHep^ Bid^−/−^ Bim^+/−^ Puma^+/+^)		
		*Bcl-xL*^*flox/flox*^ *Mcl-1*^*flox/flox*^ *Bid*^*−/−*^ *Bim*^*+/−*^ *Puma*^*+/−*^	*Bcl-xL*^*flox/flox*^ *Mcl-1*^*flox/flox*^ *Alb-Cre(+) Bid*^*−/−*^ *Bim*^*+/−*^ *Puma*^*+/−*^	6.0% (13/217)	0% (0/217)
		(Bcl-xL^+/+^ Mcl-1^+/+^ Bid^−/−^ Bim^+/−^ Puma^+/−^)	(Bcl-xL^ΔHep/ΔHep^ Mcl-1^ΔHep/ΔHep^ Bid^−/−^ Bim^+/−^ Puma^+/−^)		
		*Bcl-xL*^*flox/flox*^ *Mcl-1*^*flox/flox*^ *Bid*^*−/−*^ *Bim*^*+/−*^ *Puma*^*−/−*^	*Bcl-xL*^*flox/flox*^ *Mcl-1*^*flox/flox*^ *Alb-Cre(+) Bid*^*−/−*^ *Bim*^*+/−*^ *Puma*^*−/−*^	3.2% (7/217)	0% (0/217)
		(Bcl-xL^+/+^ Mcl-1^+/+^ Bid^−/−^ Bim^+/−^ Puma^−/−)^	(Bcl-xL^ΔHep/ΔHep^ Mcl-1^ΔHep/ΔHep^ Bid^−/−^ Bim^+/−^ Puma^−/−^)		
		*Bcl-xL*^*flox/flox*^ *Mcl-1*^*flox/flox*^ *Bid*^*−/−*^ *Bim*^*−/−*^ *Puma*^*+/+*^	*Bcl-xL*^*flox/flox*^ *Mcl-1*^*flox/flox*^ *Alb-Cre(+) Bid*^*−/−*^ *Bim*^*−/−*^ *Puma*^*+/+*^	0.9% (2/217)	0% (0/217)
		(Bcl-xL^+/+^ Mcl-1^+/+^ Bid^−/−^ Bim^−/−^ Puma^+/+^)	(Bcl-xL^ΔHep/ΔHep^ Mcl-1^ΔHep/ΔHep^ Bid^−/−^ Bim^−/−^ Puma^+/+^)		
		*Bcl-xL*^*flox/flox*^ *Mcl-1*^*flox/flox*^ *Bid*^*−/−*^ *Bim*^*−/−*^ *Puma*^*+/−*^	*Bcl-xL*^*flox/flox*^ *Mcl-1*^*flox/flox*^ *Alb-Cre(+) Bid*^*−/−*^ *Bim*^*−/−*^ *Puma*^*+/−*^	8.8% (19/217)	0% (0/217)
		(Bcl-xL^+/+^ Mcl-1^+/+^ Bid^−/−^ Bim^−/−^ Puma^+/−^)	(Bcl-xL^ΔHep/ΔHep^ Mcl-1^ΔHep/ΔHep^ Bid^−/−^ Bim^−/−^ Puma^+/−^)		
		*Bcl-xL*^*flox/flox*^ *Mcl-1*^*flox/flox*^ *Bid*^*−/−*^ *Bim*^*−/−*^ *Puma*^*−/−*^	*Bcl-xL*^*flox/flox*^ *Mcl-1*^*flox/flox*^ *Alb-Cre(+) Bid*^*−/−*^ *Bim*^*−/−*^ *Puma*^*−/−*^	1.8% (4/217)	0% (0/217)
		(Bcl-xL^+/+^ Mcl-1^+/+^ Bid^−/−^ Bim^−/−^ Puma^−/−^)	(Bcl-xL^ΔHep/ΔHep^ Mcl-1^ΔHep/ΔHep^ Bid^−/−^ Bim^−/−^ Puma^−/−^)		

Each *Alb-Cre(−)* and *Alb-Cre(+)* was expected to be born a 1:1 ratio.

We generated tamoxifen-inducible Bcl-xL^iΔHep/iΔHep^ Mcl-1^iΔHep/iΔHep^ Bid^−/−^ Bim^−/−^ Puma^−/−^ mice, in which Bcl-xL and Mcl-1 expression are nearly undetectable upon tamoxifen injection (Fig. 2F, Supplementary Fig. 3A), since the knockout of both Bcl-xL and Mcl-1 severely impairs liver development and no Bcl-xL^ΔHep/ΔHep^ Mcl-1^ΔHep/ΔHep^ Bid^−/−^ Bim^−/−^ Puma^−/−^ mice survive after birth (Table 2). After tamoxifen injection for three consecutive days, compared with control mice, Bcl-xL^iΔHep/iΔHep^ Mcl-1^iΔHep/iΔHep^ Bid^−/−^ Bim^−/−^ Puma^−/−^ mice exhibited increased serum ALT levels, elevated caspase 3/7 activity and a greater number of TUNEL-positive hepatocytes (Fig. 2G–I), and active Bax was detected in their liver tissues (Fig. 2J). All the mice in the Bcl-xL^iΔHep/iΔHep^ Mcl-1^iΔHep/iΔHep^ Bid^−/−^ Bim^−/−^ Puma^−/−^ group were euthanized within 4 days after tamoxifen injection (data not shown). On the other hand, Bcl-xL^iΔHep/iΔHep^ Mcl-1^iΔHep/iΔHep^ Bax^iΔHep/iΔHep^ Bak^−/−^ mice showed no increase in serum ALT levels after tamoxifen injection compared to those without tamoxifen injection (Supplementary Fig. 4A, B), and all of these mice survived to day 6 after tamoxifen injection (Supplementary Fig. 4C). These findings suggested that another BH3-only protein, in addition to Bid, Bim and Puma, might activate Bak/Bax and induce hepatocyte apoptosis in the absence of both Bcl-xL and Mcl-1.

### Noxa knockdown suppresses apoptosis in Bcl-xL-, Mcl-1-, Bid-, Bim- and Puma-deficient hepatocytes

**Fig. 3:**
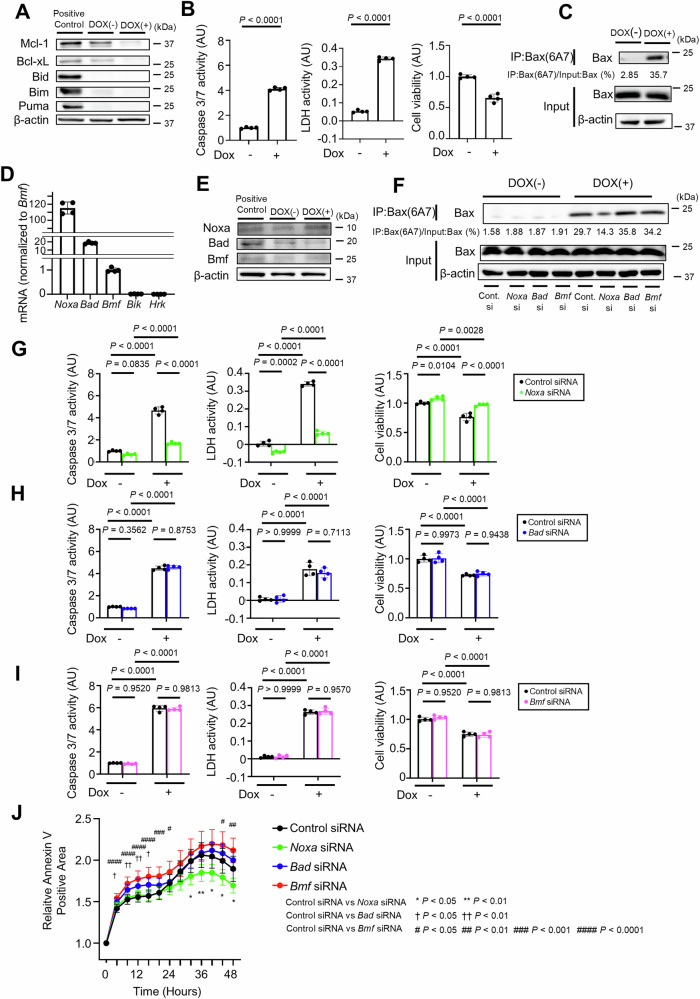
Noxa knockdown suppresses apoptosis in Bcl-xL-, Mcl-1-, Bid-, Bim- and Puma-deficient hepatocytes. **A**–**E** Immortalized *Bcl-xL*^*flox/flox*^ *Mcl-1*^*flox/flox*^ *Bid*^*−/−*^ *Bim*^*−/−*^ *Puma*^*−/−*^ mouse primary hepatocytes with doxycycline-inducible Cre recombinase were incubated with 0.3 µM doxycycline for 48 h. **A** Western blot analysis. The cell lysate of BNL.CL.2 cells was used as a positive control. **B** Caspase 3/7 activity and LDH activity in culture supernatants, cell viability assessed with a WST assay. **C** Immunoprecipitation of Bax6A7 and the ratio of Bax in the post-immunoprecipitation samples to that in the whole cell extract samples. **D** Relative mRNA expression levels of BH3-only proteins in immortalized cells. The expression levels of each protein were normalized to *Bmf* mRNA level. **E** Western blot analysis of Noxa, Bad and Bmf expression levels after doxycycline incubation. Cell lysate of BNL.CL.2 cells was used as a positive control. **F**–**J** Twenty-four hours after transfection with *Noxa, Bad, Bmf* siRNA or control siRNA, immortalized *Bcl-xL*^*flox/flox*^ *Mcl-1*^*flox/flox*^ *Bid*^*−/−*^ *Bim*^*−/−*^ *Puma*^*−/−*^ mouse primary hepatocytes were treated with 0.3 μM doxycycline for 48 h. **F** Immunoprecipitation of Bax6A7 and the ratio of Bax in the post-immunoprecipitation samples to that in the whole cell extract samples. Caspase 3/7 activity and LDH activity in the culture supernatant, cell viability assessed with a WST assay after (**G**) *Noxa*, (**H**) *Bad*, and (**I**) *Bmf* knockdown. **J** Annexin V-positive cell areas after transfection with *Noxa, Bad, Bmf* siRNA or control siRNA and doxycycline incubation. Data is represented as mean ± SD. Data was analyzed by two-tailed unpaired *t* test (**B**), One-way ANOVA with Sidak’s multiple comparisons tests (**G**–**I**) or One-way ANOVA with Dunnett’s multiple comparisons test (**J**).

To explore other BH3-only proteins involved in hepatocyte apoptosis, we generated a doxycycline-inducible Bcl-xL^iΔHep/iΔHep^ Mcl-1^iΔHep/iΔHep^ Bid^−/−^ Bim^−/−^ Puma^−/−^ hepatocyte cell line using immortalized primary mouse hepatocytes whose Bcl-xL and Mcl-1 expression was reduced 48 h after incubation with doxycycline (Fig. 3A). Caspase 3/7 activity and LDH activity were significantly increased, and relative cell viability was significantly decreased after incubation with doxycycline (Fig. 3B). Active Bax was also detected after doxycycline treatment (Fig. 3C). Among the five remaining BH3-only proteins, *Noxa*, *Bad*, and *Bmf* were expressed in this immortalized cell line, whereas *Bik* and *Hrk* were not detected (Fig. 3D). *Bik* and *Hrk* were also not detected in wild-type murine primary hepatocytes (Supplementary Fig. 5A). After doxycycline treatment, only Noxa expression increased among Noxa, Bad and Bmf (Fig. 3E). SiRNA-mediated knockdown of *Noxa* led to a significant reduction in Bax activation, Caspase 3/7 activity, LDH activity, and Annexin V-positive areas and a significant increase in relative cell viability in doxycycline-treated Bcl-xL^iΔHep/iΔHep^ Mcl-1^iΔHep/iΔHep^ Bid^−/−^ Bim^−/−^ Puma^−/−^ cells (Fig. 3F, G, J; Supplementary Fig. 5B), whereas siRNA-mediated knockdown of *Bad* or *Bmf* did not (Fig. 3F, H–J; Supplementary Fig. 5C, D).

### Disruption of Noxa decreases hepatocyte apoptosis in Bcl-xL Mcl-1-, Bid-, Bim- and Puma-deficient mice and Mcl-1-deficient mice

**Fig. 4:**
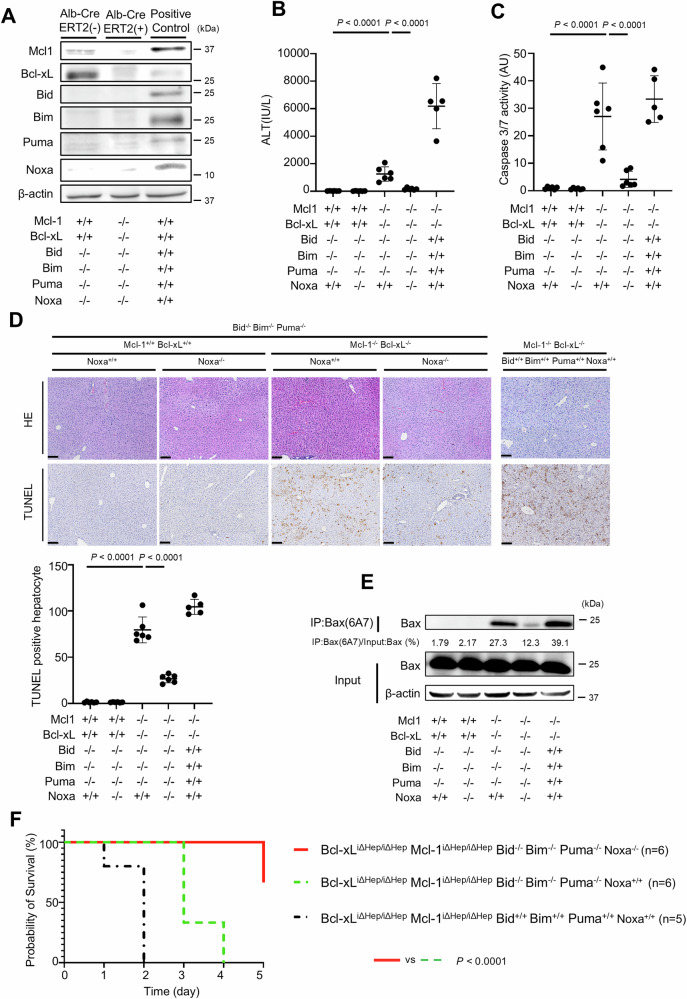
Additional disruption of Noxa significantly decreases hepatocyte apoptosis in Bcl-xL-, Mcl-1-, Bid-, Bim- and Puma-deficient mice. We generated *Bcl-xL*^*flox/flox*^ *Mcl-1*^*flox/flox*^ *Alb-Cre ERT2 Bid*^*−/−*^ *Bim*^*−/−*^ *Puma*^*−/−*^ *Noxa*^*−/−*^ mice via CRISPR/Cas9 technology. These mice were injected intraperitoneally with 1 mg of tamoxifen and sacrificed after 12 h; n = 6 per group. The data of *Bcl-xL*^*flox/flox*^ *Mcl-1*^*flox/flox*^ *Alb-Cre ERT2* mice are presented as controls; *n* = 5 (**B**–**F**). **A** Expression of Mcl-1, Bcl-xL, Bid, Bim, Puma, Noxa and β-actin proteins were assessed via Western blotting analysis of liver lysates after tamoxifen injection. C57BL/6J mouse was used as a positive control. **B**, **C** Serum ALT levels and Caspase 3/7 activity. **D** Representative images of H&E staining and TUNEL staining and their quantitative results. **E** Immunoprecipitation of Bax6A7 and the ratio of Bax in the post-immunoprecipitation samples to that in the whole cell extract samples. **F** Probability of survival after consecutive tamoxifen injections (*n* = 6). Data is represented as mean ± SD. Data was analyzed by One-way ANOVA with Sidak’s multiple comparisons tests (**B**–**D**) or the log-rank test (**F**). Scale bar (**D**): 100 μm.

We next examined the role of Noxa in vivo. We disrupted Noxa via CRISPR-Cas9 technology to generate tamoxifen-inducible Bcl-xL^iΔHep/iΔHep^ Mcl-1^iΔHep/iΔHep^ Bid^−/−^ Bim^−/−^ Puma^−/−^ Noxa^−/−^ mice (Supplementary Fig. 6A, B). Bcl-xL and Mcl-1 expression in the liver sections of these mice was nearly undetectable at 12 h after tamoxifen injection (Fig. 4A, Supplementary Fig. 7A). Disruption of Noxa significantly reduced the serum ALT level and caspase 3/7 activity in tamoxifen-treated Bcl-xL^iΔHep/iΔHep^ Mcl-1^iΔHep/iΔHep^ Bid^−/−^ Bim^−/−^ Puma^−/−^ mice (Fig. 4B, C). The number of TUNEL-positive hepatocytes was significantly decreased (Fig. 4D), and Bax activation was suppressed by Noxa disruption (Fig. 4E). Although some Bcl-xL^iΔHep/iΔHep^ Mcl-1^iΔHep/iΔHep^ Bid^−/−^ Bim^−/−^ Puma^−/−^ Noxa^−/−^ mice died 5 days after tamoxifen injection, Noxa disruption significantly prolonged their survival (Fig. 4F).

**Fig. 5:**
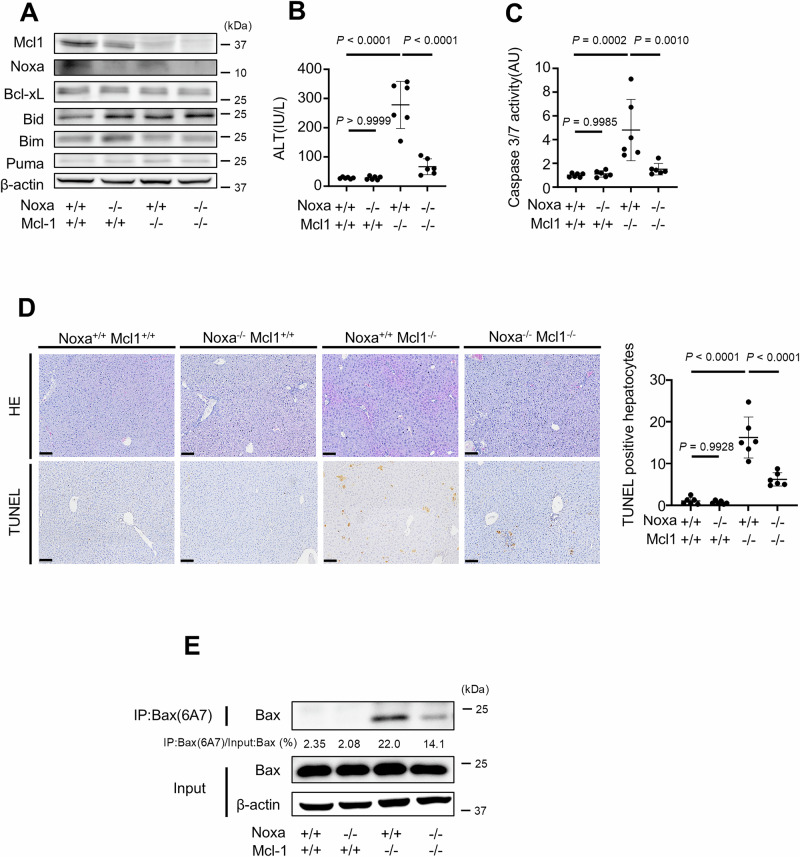
Disruption of Noxa suppresses hepatocyte apoptosis in hepatocyte-specific Mcl-1 knockout mice. We generated *Mcl-1*^*flox/flox*^ *Alb-Cre Noxa*^*−/−*^ mice and control mice. These mice were analyzed at 6 to 8 weeks of age; *n* = 6 per group. **A** Expressions of Mcl-1, Noxa, Bcl-xL, Bid, Bim, Puma and β-actin proteins were assessed via Western blot analysis. **B**, **C** Serum ALT levels and Caspase 3/7 activity. **D** Representative images of H&E staining and TUNEL staining and their quantitative results. **E** Immunoprecipitation of Bax6A7 and the ratio of Bax in the post-immunoprecipitation samples to that in the whole cell extract samples. Data is represented as mean ± SD. Data was analyzed by One-way ANOVA with Sidak’s multiple comparisons tests (**B**–**D**). Scale bar (**D**): 100 μm.

To discern the potential contribution of Noxa to hepatocyte apoptosis, particularly in the presence of Bid, Bim and Puma, we knocked out Noxa in Mcl-1^ΔHep/ΔHep^ mice (Fig. 5A). Compared with Noxa+/+ mice, Noxa^−/−^ mice showed no differences in serum ALT levels, serum caspase 3/7 activity or the number of TUNEL-positive cells (Fig. 5B–D). Compared with Mcl-1^ΔHep/ΔHep^ mice, Mcl-1^ΔHep/ΔHep^ Noxa^−/−^ mice exhibited significantly lower serum ALT levels and serum caspase 3/7 activity as well as fewer TUNEL-positive hepatocytes in liver sections (Fig. 5B–D). Mcl-1^ΔHep/ΔHep^ Noxa^−/−^ mice also presented lower expression levels of active Bax in liver tissues (Fig. 5E).

### No BH3-only proteins other than Bid, Bim, Puma and Noxa contribute to hepatocyte apoptosis caused by the deletion of both Mcl-1 and Bcl-xL

**Fig. 6:**
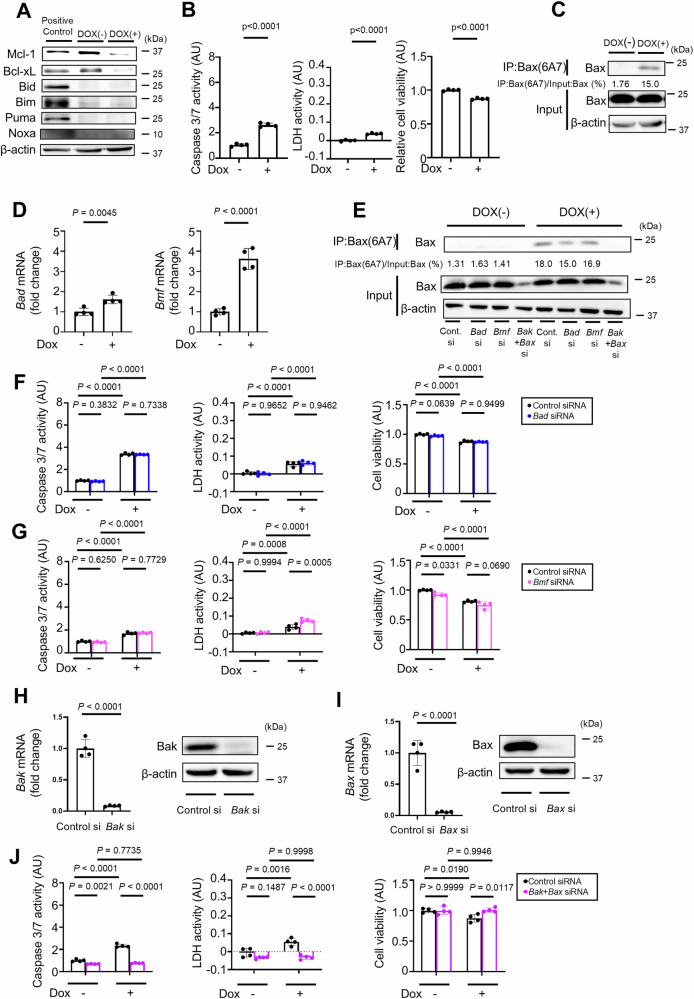
No BH3-only proteins other than Bid, Bim, Puma and Noxa contribute to hepatocyte apoptosis caused by the deletion of both Mcl-1 and Bcl-xL. **A**–**D** Immortalized *Bcl-xL*^*flox/flox*^ *Mcl-1*^*flox/flox*^ *Bid*^*−/−*^ *Bim*^*−/−*^ *Puma*^*−/−*^ *Noxa*^*−/−*^ mouse primary hepatocytes with doxycycline-inducible Cre recombinase were incubated with 0.3 µM doxycycline for 48 h. **A** Western blot analysis. The cell lysate of BNL.CL.2 cells was used as a positive control. **B** Caspase 3/7 activity and LDH activity in culture supernatants, cell viability assessed with a WST assay. **C** Immunoprecipitation of Bax6A7 and the ratio of Bax volume in post-immunoprecipitation samples to that in input samples. **D** Relative mRNA expression levels of *Bad* and *Bmf* after incubation with doxycycline. **E**–**J** Immortalized *Bcl-xL*^*flox/flox*^ *Mcl-1*^*flox/flox*^ *Bid*^*−/−*^ *Bim*^*−/−*^ *Puma*^*−/−*^ *Noxa*^*−/−*^ mouse primary hepatocytes were treated with 0.3 μM doxycycline for 48 hours after transfection with *Bad*, *Bmf, Bak* and *Bax* siRNA or control siRNA. **E** Immunoprecipitation of Bax6A7 and the ratio of Bax in the post-immunoprecipitation samples to that in the whole cell extract samples. Caspase 3/7 activity and LDH activity in culture supernatants, WST assay after (**F**) *Bad* and (**G**) *Bmf* knockdown. Relative mRNA expression levels and Western blotting of (**H**) *Bak* and (**I**) *Bax* after siRNA transfection. **J** Caspase 3/7 activity and LDH activity in culture supernatants, WST assay after *Bak* and *Bax* knockdown. Data is represented as mean ± SD. Data was analyzed by two-tailed unpaired *t* tests (**B**, **D**, **H**, **I**) or One-way ANOVA with Sidak’s multiple comparisons tests (**F**, **G**, **J**).

To examine the role of the remaining BH3-only proteins in Bak/Bax-dependent hepatocyte apoptosis, we generated a doxycycline-inducible Bcl-xL^iΔHep/iΔHep^ Mcl-1^iΔHep/iΔHep^ Bid^−/−^ Bim^−/−^ Puma^−/−^ Noxa^−/−^ hepatocyte cell line using immortalized primary mouse hepatocytes. Caspase 3/7 activity and LDH activity were significantly increased, and relative cell viability was significantly decreased after incubation with doxycycline (Figs. 6A, B). The active form of Bax was still detected after doxycycline treatment (Fig. 6C). Among the four remaining BH3-only proteins, *Bad* and *Bmf* were expressed in Bcl-xL^iΔHep/iΔHep^ Mcl-1^iΔHep/iΔHep^ Bid^−/−^ Bim^−/−^ Puma^−/−^ Noxa^−/−^ cells. After doxycycline treatment, *Bad* and *Bmf* mRNA expression levels increased (Fig. 6D). However, siRNA-mediated knockdown of either of these genes did not affect Bax activation, caspase 3/7 activity, LDH activity and relative cell viability in doxycycline-treated Bcl-xL^iΔHep/iΔHep^ Mcl-1^iΔHep/iΔHep^ Bid^−/−^ Bim^−/−^ Puma^−/−^ Noxa^−/−^ cells (Fig. 6E–G). On the other hand, siRNA-mediated knockdown of both *Bak* and *Bax* completely abrogated the increases in caspase 3/7 activity and LDH activity and decreased cell viability (Fig. 6H–J). The active form of Bax was also reduced by *Bak* and *Bax* knockdown (Fig. 6E).

To explore other molecules that are involved in Bak/Bax-dependent apoptosis in the absence of Bid, Bim, Puma and Noxa, outside the realm of BH3-only proteins, we performed RNA-seq of a doxycycline-induced Bcl-xL^iΔHep/iΔHep^ Mcl-1^iΔHep/iΔHep^ Bid^−/−^ Bim^−/−^ Puma^−/−^ Noxa^−/−^ immortalized hepatocyte cell line and a doxycycline-induced Bcl-xL^iΔHep/iΔHep^ Mcl-1^iΔHep/iΔHep^ Bid^−/−^ Bim^−/−^ Puma^−/−^ Noxa^+/+^ immortalized hepatocyte cell line (Figs. 3A and 6A). RNA-seq analysis revealed 977 genes in Bcl-xL^iΔHep/iΔHep^ Mcl-1^iΔHep/iΔHep^ Bid^−/−^ Bim^−/−^ Puma^−/−^ Noxa^−/−^ hepatocytes with FPKM values greater than twice those in Bcl-xL^iΔHep/iΔHep^ Mcl-1^iΔHep/iΔHep^ Bid^−/−^ Bim^−/−^ Puma^−/−^ Noxa^+/+^ hepatocytes (Supplementary Table 1). Among these genes, no Bcl-2 family proteins were detected, while *Caspase-6*, *Caspase-9* and *Caspase-12*, which are associated with apoptosis [29, 30], were detected. In the present study, we focused on *caspase-6*, which is known as an executor caspase, as well as caspase3/caspase7. SiRNA-mediated knockdown of *caspase-6* slightly but significantly increased relative cell viability in doxycycline-treated Bcl-xL^iΔHep/iΔHep^ Mcl-1^iΔHep/iΔHep^ Bid^−/−^ Bim^−/−^ Puma^−/−^ Noxa^−/−^ cells, while it did not affect caspase 3/7 activity (Supplementary Fig. 8A, B). Similarly, siRNA-mediated knockdown of *caspase-6* slightly increased the relative viability of BNL.CL.2 cells, a murine hepatocyte cell line, treated with ABT-737, a Bcl-xL inhibitor that induces Bak/Bax-dependent hepatocyte apoptosis [31], without affecting caspase 3/7 activity (Supplementary Fig. 9A).

## Discussion

Our previous studies revealed that the BH3-only proteins Bid and Bim are involved in the execution of Bax/Bak-dependent apoptosis due to the loss of anti-apoptotic molecules such as Bcl-xL and Mcl-1 in hepatocytes [22]. In the present study, we investigated the functions of other members of the same family of molecules and reported that Puma and Noxa have similar functions.

We demonstrated that disruption of Puma attenuated hepatocyte apoptosis and suppressed Bax activation induced by the absence of Bcl-xL and Mcl-1 in hepatocytes (Fig. 2A–E). These findings suggest that Puma is involved in the execution of Bax/Bak-dependent hepatocyte apoptosis.

When BH3-only proteins, Bid, Bim and Puma were eliminated, additional Noxa knockout mitigated hepatocyte apoptosis and suppressed Bax activation induced by the simultaneous deletion of Bcl-xL and Mcl-1 (Fig. 4A–E). We also demonstrated that disruption of Noxa in the presence of Bid, Bim and Puma suppressed hepatocyte apoptosis and Bax activation induced by the absence of Mcl-1 (Fig. 5A–E). Our findings suggest that Noxa may have a similar function as Puma and may be involved in inducing hepatocyte apoptosis via Bak/Bax activation in the absence of anti-apoptotic Bcl-2 family proteins. Some previous studies in vitro and in mouse embryonic fibroblasts (MEFs) reported that Puma and Noxa can activate Bak/Bax [12, 32–34], and our findings seem to be consistent with these reports.

Among the five anti-apoptotic molecules, Bcl-w was also expressed in hepatocytes in addition to Bcl-xL and Mcl-1 (Fig. 1A, B). However, previous reports have shown that, in contrast to Bcl-xL or Mcl-1, the knockout of Bcl-w did not result in a phenotype in hepatocytes [35, 36]. Therefore, the anti-apoptotic members of the Bcl-2 family that function in hepatocytes in vivo are likely to be Bcl-xL and Mcl-1.

In the present study, we demonstrated that the loss of Bim, Bid, Puma, and Noxa cooperatively attenuated apoptosis induced by the absence of both Bcl-xL and Mcl-1 (Fig. 4A–E). These results suggest that these molecules may directly activate Bak or Bax without the intervention of the anti-apoptotic Bcl-2 family proteins. However, our system of knocking out all the Bcl-xL and Mcl-1 genes in mice is only a drug-inducible system because the mice would be otherwise lethal (Tables 1 and 2), and we cannot rule out the possibility that trace amounts of anti-apoptotic proteins remain. Therefore, we cannot exclude the possibility that BH3-only proteins, Bid, Bim, Puma, and/or Noxa, caused apoptosis by inhibiting the function of the trace amount of remaining anti-apoptotic proteins; therefore, knocking out these proteins might attenuate apoptosis. As for Noxa, it has been reported that its main role was a kind of sensitizer that induces apoptosis by forming a complex with Mcl-1 and degrading it [37–39]. However, since loss of Noxa attenuated hepatocyte apoptosis in nondrug-induced hepatocyte-specific Mcl-1 knockout mice (Fig. 5A–E), there may be another mechanism for the apoptosis-inducing function of Noxa.

In the present study, simultaneous loss of Bid, Bim, Puma and Noxa in anti-apoptotic proteins deficient mice markedly suppressed apoptosis in hepatocytes (Fig. 4A–E) but failed to completely arrest apoptosis (Fig. 6A–C). Among the BH3-only proteins other than Bid, Bim, Puma and Noxa, Bad and Bmf seemed not to be involved in hepatocyte apoptosis in the absence of anti-apoptotic proteins (Fig. 6E–G). Bik and Hrk are likely not involved in Bak/Bax-dependent apoptosis in hepatocytes, likely because of their undetectable expression levels (Fig. 3D, Supplementary Fig. 5A). These results suggest that the absence of all BH3-only proteins does not halt Bak/Bax-dependent hepatocyte apoptosis caused by Bcl-xL and Mcl-1 deficiency, suggesting the existence of a BH3-only proteins-independent Bak/Bax activation mechanism, that is, the previously reported mechanism by which the mitochondrial outer membrane activates Bax [17]. At the same time, we cannot exclude the possibility that there may be unidentified BH3-only proteins that directly activate Bak/Bax.

To further explore additional molecules involved in hepatocyte apoptosis beyond Bid, Bim, Puma and Noxa, we analyzed the RNA-seq data and focused on caspase-6 in the present study (Supplementary Table 1). We showed that the knockdown of caspase-6 increased relative cell viability in Bid^−/−^ Bim^−/−^ Puma^−/−^ Noxa^−/−^ cells after the simultaneous deletion of Bcl-xL and Mcl-1 (Supplementary Fig. 8A, B). It has been reported that caspase-6 mediates a positive feedback loop to sustain the caspase cascade in hepatocytes via the AMPK-caspase-6 pathway in nonalcoholic steatohepatitis [40], although this feedback loop is not consistent with our findings because caspase-6 knockdown had no effect on caspase-3/7 activity (Supplementary Figs. 8B and 9A). Another report indicated that caspase-3, 6 and 7 have nonredundant roles and that caspase-6 operates downstream of caspase-3, contributing partially to the induction of apoptosis [41]. The results of our study align with these findings. Even though our RNA-seq analysis did not reveal other candidates for unidentified BH3-only proteins, further investigation is required to determine whether caspase-6, and other unidentified BH3-only proteins, regulate Bak/Bax-dependent hepatocyte apoptosis.

A limitation of this study is that there may be little leakage of the Cre^ERT2^ system because the expression levels of Bcl-xL and Bax were low in Bcl-xL^iΔHep/iΔHep^ Mcl-1^iΔHep/iΔHep^ Bax^iΔHep/iΔHep^ Bak^−/−^ mice even without tamoxifen injection (Supplementary Fig. 4B), which might lead to higher serum ALT levels in Bcl-xL^iΔHep/iΔHep^ Mcl-1^iΔHep/iΔHep^ Bax^iΔHep/iΔHep^ Bak^−/−^ mice without tamoxifen than in Bcl-xL^+/+^ Mcl-1^+/+^ Bax^+/+^ Bak^−/−^ mice (Supplementary Fig. 4A).

In conclusion, our present study revealed that BH3-only proteins, Puma and Noxa, as well as Bid and Bim, are involved in the execution of Bax/Bak-dependent apoptosis caused by the deletion of anti-apoptotic molecules in hepatocytes. No BH3-only proteins other than Bid, Bim Puma and Noxa are involved in this mechanism. Furthermore, our data suggest the existence of a BH3-only proteins-independent Bak/Bax activation mechanism. Understanding the orchestration of these Bcl-2 family proteins in hepatocytes provides insights into hepatocyte homeostasis and the pathogenesis of hepatocyte apoptosis.

## Materials and methods

### Mice

Mice carrying a Bcl-x gene with two loxP sequences at the promoter region and a second intron (*Bcl-x*^*flox/flox*^), mice carrying an Mcl-1 gene encoding amino acids 1 through 179 flanked by two loxP sequences (*Mcl-1*^*flox/flox*^), and heterozygous Alb-Cre transgenic mice expressing the Cre recombinase gene under the control of the albumin gene promoter have been described previously [20, 42, 43]. Hepatocyte-specific Bcl-xL knockout mice (*Bcl-xL*^*flox/flox*^ *Alb-Cre*; Bcl-xL^ΔHep/ΔHep^ mice) were generated as described previously [20] (129SvEv and C57BL/6 mixed background). Hepatocyte-specific Mcl-1 knockout mice *(Mcl-1*^*flox/flox*^ *Alb-Cre*; Mcl-1^ΔHep/ΔHep^ mice) have been described previously [28] (C57BL/6 background).

Puma knockout mice [44] (*Puma*^*−/−*^; Puma^−/−^ mice, Strain #011067) and Bim knockout mice [45] (*Bim*^*−/−*^; Bim^−/−^ mice, Strain #004525) were purchased from the Jackson Laboratory (Bar Harbor, ME, USA). Bid knockout mice (*Bid*^*−/−*^; Bid^−/−^ mice) were generated as described previously [46] (C57BL/6 background). Bak/Bax double knockout mice (*Bax* ^*flox/flox*^ *Alb-Cre Bak*^*−/−*^; Bax^ΔHep/ΔHep^ Bak^−/−^ mice, Strain #006329) were purchased from the Jackson Laboratory and generated as described previously [19, 47]. Bcl-xL^ΔHep/ΔHep^ mice and Mcl-1^ΔHep/ΔHep^ mice were crossed with Puma^−/−^ mice to produce Bcl-xL^ΔHep/ΔHep^ Puma^−/−^ mice and Mcl-1^ΔHep/ΔHep^ Puma^−/−^ mice, respectively. Mated *Bcl-xL*^*flox/flox*^ *Alb-Cre Puma*^*+/−*^ and *Bcl-xL*^*flox/flox*^ *Puma*^*+/−*^, *Mcl-1*^*flox/flox*^ *Alb-Cre Puma*^*+/−*^ and *Mcl-1*^*flox/flox*^ *Puma*^*+/−*^ offspring were analyzed at 6 to 8 weeks of age. We further crossed Bcl-xL^ΔHep/ΔHep^ mice, Mcl-1^ΔHep/ΔHep^ mice, Bid^−/−^ mice, Bim^−/−^ mice and Puma^−/−^ mice to generate Bcl-xL^ΔHep/ΔHep^ Mcl-1^ΔHep/+^ Bid^−/−^ Bim^−/−^ Puma^−/−^ mice. The offspring of *Bcl-xL*^*flox/flox*^ *Mcl-1*^*flox/+*^ *Alb-Cre Bid*^*−/−*^ *Bim*^*−/−*^ (or *Bim*^*+/−*^) *Puma*^*+/−*^ mice and *Bcl-xL*^*flox/flox*^ *Mcl-1*^*flox/flox*^ *Bid*^*−/−*^ *Bim*^*−/−*^ (or *Bim*^*+/−*^) *Puma*^*+/−*^ mice were analyzed at 6 to 8 weeks of age. Of note, for this mating, we used *Bim*^*−/−*^ male and *Bim*^*+/−*^ female mice because *Bim*^*−/−*^ female mice are infertile. We also crossed Bcl-xL^ΔHep/ΔHep^ mice and Mcl-1^ΔHep/ΔHep^ mice with Bax^ΔHep/ΔHep^ Bak^−/−^ mice to generate Bcl-xL^ΔHep/ΔHep^ Mcl-1^ΔHep/+^ Bax^ΔHep/ΔHep^ Bak^−/−^ mice. The offspring of the *Bcl-xL*^*flox/flox*^ *Mcl-1*^*flox/+*^ *Bax* ^*flox/flox*^ *Alb-Cre Bak*^*+/−*^ mice and the *Bcl-xL*^*flox/flox*^ *Mcl-1*^*flox/flox*^ *Bax* ^*flox/flox*^ *Bak*^*+/−*^ mice were analyzed at 6 to 8 weeks of age. Tamoxifen-inducible hepatocyte-specific Cre mice (*Albumin-Cre-ERT2* mice) were kindly provided by Professor Pierre Chambon [48] (C57BL/6 background), and we generated tamoxifen-inducible hepatocyte-specific Bcl-xL and Mcl-1 knockout mice (*Bcl-xL*^*flox/flox*^ *Mcl-1* ^*flox/flox*^*Alb-Cre*^*ERT2*^; Bcl-xL^iΔHep/iΔHep^ Mcl-1^iΔHep/iΔHep^ mice) and Bcl-xL^iΔHep/iΔHep^ Mcl-1^iΔHep/iΔHep^ Bid^−/−^ Bim^−/−^ Puma^−/−^ mice by crossing them. We also generated Bcl-xL^iΔHep/iΔHep^ Mcl-1^iΔHep/iΔHep^ Bax^iΔHep/iΔHep^ Bak^−/−^ mice by crossing them with Bax^ΔHep/ΔHep^ Bak^−/−^ mice. The mice were injected intraperitoneally with 1 mg of tamoxifen (Sigma‒Aldrich, St. Louis, MO, USA) at the indicated times. For all the animal experiments, individual mice were allocated to different experimental groups on the basis of their genotypes. For mouse sacrifice as well as post-sacrifice analysis, such as quantification by TUNEL staining, investigators were blinded. The animals were housed in cages under specific pathogen-free conditions with free access to water and standard mouse chow.

### Generation of Noxa knockout mice

Fertilized eggs from *Bcl-xL*^*flox/flox*^ *Mcl-1*^*flox/flox*^ *Alb-Cre*^*ERT2*^ *Bid*^*−/−*^ *Bim*^*+/−*^ *Puma*^*−/−*^ *mice* were produced and used for in vitro fertilization. Subsequently, Noxa knockout guide RNA (gRNA) and Cas9 proteins were introduced into these fertilized eggs through electroporation. gRNA was designed using a software tool (http://crispor.tefor.net/andhttps://crispr.dbcls.jp/) to predict unique target sites throughout the mouse genome. Cas9 proteins were obtained from Alt-R^®^ S.p.Cas9 Nucleases 3NLS (Integrated DNA Technologies, Inc. Coralville, Iowa, USA). Deletion of the Noxa sequence was confirmed by genotyping DNA isolated from the resulting offspring (Supplementary Fig. 6A, B). We generated Bcl-xL^iΔHep/iΔHep^ Mcl-1^iΔHep/iΔHep^ Bid^−/−^ Bim^−/−^ Puma^−/−^ Noxa^−/−^ mice by crossing *Bcl-xL*^*flox/flox*^ *Mcl-1*^*flox/flox*^ *Alb-Cre*^*ERT2*^ *Bid*^*−/−*^ *Bim*^*−/−*^ *(or Bim*^*+/−*^*) Puma*^*−/−*^ *Noxa*^*+/−*^ mice with *Bcl-xL*^*flox/flox*^ *Mcl-1*^*flox/flox*^ *Bid*^*−/−*^ *Bim*^*+/−*^ *(or Bim*^*−/−*^*) Puma*^*−/−*^ *Noxa*^*+/−*^ mice and analyzed their offspring.

### Quantitative real-time RT‒PCR analysis

Total RNA was extracted from cells and liver tissues via an RNeasy Kit (QIAGEN, Hilden, Germany) and reverse transcribed via ReverTra Ace qPCR RT Master Mix (TOYOBO, Osaka, Japan). Real-time RT‒PCR was performed via TaqMan gene expression assays with an HT7900 Fast Real-Time PCR System (Thermo Fisher Scientific, Waltham, MA, USA). The following TaqMan gene expression primers were used: *Noxa* (Mm00451763_m1), *Bad* (Mm00432042_m1), *Bmf* (Mm00506773_m1), *Bik* (Mm00476123_m1), *Hrk* (Mm01208086_m1), *Bax* (Mm00432051_m1), *Bak* (Mm00432045_m1), *Caspase6* (Mm01321726_g1) and *Actb* (Mm02619580_g1). Gene expression levels were normalized to those of *Actb*.

### Western blotting analysis

Whole-cell extracts and liver tissues were lysed in lysis buffer (1% Nonidet P-40, 0.5% sodium deoxycholate, and phosphate-buffered saline, pH 7.4; 0.1% sodium dodecyl sulfate; 1× proteinase inhibitor cocktail (Nacalai Tesque, Kyoto, Japan); and 1× phosphatase inhibitor cocktail (Nacalai Tesque)). The supernatant was collected via centrifugation (13,500 × *g* for 30 min at 4 °C), and the protein concentrations were determined via a bicinchoninic acid protein assay kit (Thermo Fisher Scientific). Protein samples of equal concentration were separated on sodium dodecyl sulfate polyacrylamide gels and transferred onto polyvinylidene fluoride membranes. For immunodetection, the following antibodies were used: an anti-Bid antibody (#2003), anti-Bim antibody (#2933), anti-Puma antibody (#24633), anti-Bak antibody (#3814), anti-Bax antibody (#2772), anti-Bcl-w antibody (#2724), anti-Bcl-2 antibody (#2876), anti-Bad antibody (#9292), and anti-Caspase6 antibody (#9762) purchased from Cell Signaling Technology (Beverly, MA, USA); an anti-Mcl-1 antibody (600-401-394) purchased from Rockland Immunochemicals (Limerick, PA, USA); an anti-Bcl-xL antibody (sc-634) purchased from Santa Cruz Biotechnology (Dallas, TX, USA); an anti-Noxa antibody (ab23563), anti-Bcl-2A1 antibody (ab45413) and anti-Bmf antibody (ab9655) purchased from Abcam (Cambridge, MA, USA); and an anti-β-actin antibody (A5316) purchased from Sigma‒Aldrich. All of full and uncropped western blots are demonstrated in the supplementary material.

### Small interfering RNA (siRNA)-mediated knockdown

Immortalized cells were transfected with 10 nM siRNA via Lipofectamine RNAiMAX (Thermo Fisher Scientific) according to the manufacturer’s protocol. The cells were treated with 0.3 µM doxycycline (Wako Pure Chemical Industries, Osaka, Japan) for 24 h and then analyzed 72 h after transfection. The following siRNAs were used: siRNA against *Noxa* (s81669), siRNA against *Bad* (s233500), siRNA against *Bmf* (s101192), siRNA against *Bak* (s62860), siRNA against *Bax* (s62874), and siRNA against *Caspase6* (s63387). The appropriate negative controls were purchased from Thermo Fisher Scientific.

### Hepatic function, caspase activity, and hepatocyte death analyses

Serum alanine transaminase (ALT) levels were measured via a DRI-CHEM NX700iV (FUJIFILM, Tokyo, Japan). Serum caspase 3/7 activity was measured with a luminescent substrate assay for caspase3 and caspase7 (Caspase-Glo Assay, Promega, Madison, WI, USA) according to the manufacturer’s protocol and is shown as relative values. Liver sections were stained with hematoxylin and eosin (HE). To detect apoptotic hepatocytes, terminal deoxynucleotidyl transferase-mediated deoxyuridine triphosphate nick-end labeling (TUNEL) staining was performed with an ApopTag Kit (Millipore, Moldheim, France) according to the manufacturer’s protocol. TUNEL-positive cells were counted in four fields per liver section, and the average number of TUNEL-positive cells was determined.

### Immunofluorescence staining

Fresh-frozen sections of liver samples were prepared for staining, and immunofluorescence staining was performed according to the manufacturer’s protocol. For immunodetection, the following primary antibodies were used: an anti-Mcl-1 antibody (Santa Cruz Biotechnology, sc-377487) and an anti-Bcl-xL antibody (Cell Signaling Technology, #2764). The following secondary antibodies were purchased from Abcam: goat anti-mouse IgG H&L (Alexa Fluor® 488) preadsorbed (ab150117) and goat anti-rabbit IgG H&L (Alexa Fluor® 647) preabsorbed (ab150083). Fluorescence images were analyzed with FV1200 (Olympus Life Science, Tokyo, Japan).

### In vitro cell death assay

Cell viability was measured via a water-soluble tetrazolium salt (WST) assay (Nakalai Tesque), and the results are shown as relative values. Caspase 3/7 activity was measured with a luminescent substrate assay for caspase3 and caspase7 (Promega) according to the manufacturer’s protocol and is shown as relative values. The LDH activity of cultured cells was measured via a Cytotoxicity LDH Assay Kit-WST (Dojindo Laboratories, Kumamoto, Japan), and the results are presented as relative values. ABT-737 was purchased from Selleck Chemicals (Houston, TX, USA). An IncuCyte SX1 live-cell analysis system (Sartorius Japan, Tokyo, Japan) was used to analyze the apoptosis of immortalized cells cultured with doxycycline. Twenty-four hours after siRNA-mediated knockdown, Annexin V Green Reagent for Apoptosis (Sartorius, Japan) was added to the culture medium of the immortalized cells at the same time as doxycycline. Images of Annexin V-positive areas at different time points were obtained, and the fluorescence intensity was normalized to that of the Annexin V-positive areas at 0 h. All of the images were analyzed via IncuCyte 2021A software (Sartorius Japan).

### Immunoprecipitation of Bax 6A7

The method for immunoprecipitation of Bax 6A7 was described previously [49]. Whole-cell extracts and liver tissue homogenate were subjected to immunoprecipitation for Bax6A7, followed by immunoblotting for total BAX. They were lysed in 1% CHAPS lysis buffer (150 mM NaCl, 10 mM HEPES, pH 7.4, 1% CHAPS) supplemented with proteinase inhibitor (Nacalai Tesque). To preclear the sample, 600 µg of cell lysate or 1000 µg of liver tissue homogenate was collected and incubated with 12 µl of 50% slurry protein G agarose beads (Santa Cruz Biotechnology, sc-2002) for 30 min at 4 °C on a rotator. The precleared samples were then incubated with 5 µl of Bax6A7 antibody (Santa Cruz Biotechnology, sc-23959) overnight at 4 °C on a rotator. 20 µl of 50% slurry protein G agarose beads (Santa Cruz Biotechnology) were added to the samples the next day and incubated for 2 h at 4 °C on a rotator. The beads were collected, washed with lysis buffer three times and boiled for 15 min in 50 µl of 1X sample buffer. An equal amount of protein from solubilized whole cell extracts or liver tissue homogenates were prepared. Post-immunoprecipitation samples using Bax6A7, as well as whole cell or liver tissue samples, were then analyzed by immunoblotting for total BAX. They were separated on the same sodium dodecyl sulfate polyacrylamide gels and transferred onto polyvinylidene fluoride membranes. The membranes were blocked in PBS containing 5% milk for 1 h and incubated with primary antibodies overnight at 4 °C. For immunodetection, the following primary antibodies were used: total Bax (Cell Signaling Technology, #2772) and β-actin (Sigma‒Aldrich, A5316). The next day, the membranes were incubated with an anti-rabbit IgG (NA934V, Cytiva, Tokyo, Japan, 1:1000) or an anti-mouse IgG (NA931V, Cytiva 1:1000) secondary antibody. For protein visualization, Fusion Solo S (Vilber, Marne-la-Vallée, France) was used. Membrane images were captured with the same exposure time to compare Bax expression in the post-immunoprecipitation samples using Bax6A7 antibody with that in the whole cell extract samples. The ratio of Bax in the post-immunoprecipitation samples to that in the whole cell extract samples was calculated and displayed in the corresponding Western blot image.

### Cell culture, primary hepatocyte isolation and immortalization

Mouse primary hepatocytes were isolated via the two-step collagenase‒pronase liver perfusion method in the same manner as previously reported [31]. Isolated hepatocytes were cultured in William’s eagle medium (Thermo Fisher Scientific) supplemented with 10% fetal bovine serum (FCS), 2 mM L-glutamine (Thermo Fisher Scientific), 100 nM insulin (Sigma‒Aldrich) and 100 nM dexamethasone (Sigma‒Aldrich). To immortalize mouse primary hepatocytes, they were transfected with a lentiviral vector expressing simian virus 40 large T antigen (SV40T) purchased from Addgene (Watertown, MA, USA, plasmid #22298). BNL CL.2 cells were purchased from the American Type Culture Collection (ATCC, Manassas, VA, USA). Hep-55.1C cells were purchased from Cytion (Eppelheim, Germany). The cell lines were tested for mycoplasma contamination yearly and as needed.

These cells were cultured in Dulbecco’s modified Eagle’s medium (DMEM; Sigma‒Aldrich) in an incubator with 5% CO_2_ at 37 °C. The medium contained 10% fetal bovine serum and 1% antibiotics (Anti-Anti; Thermo Fisher).

### Generation of immortalized cells with doxycycline-dependent Cre/LoxP recombination

The resulting plasmid (pLenti-Cre-IRES-PuroR, Addgene #30205) was amplified via PCR and electrophoresed on a 0.8% gel. The 1029 bp band was eluted from the gel and used as an insert. The vector (pLenti-iCas9-neo, Addgene #85400) was cleaved by two restriction enzymes, XhoI and BsmBI (Bio-Rad, Hercules, CA, USA), and electrophoresed on a 0.8% gel. The 9959 bp band was eluted and used as a backbone. Using an In-Fusion cloning kit (Takara Bio Inc., Shiga, Japan) with these inserts and backbones, a plasmid with doxycycline-inducible Cre and neomycin resistance gene constructs (pLenti-iCre-neo) was prepared. The obtained plasmids were transfected into HEK293 cells for lentivirus packaging. The viral supernatant was used to transfect the immortalized primary hepatocytes. These cells were cultured with 700 μg/ml neomycin (Thermo Fisher Scientific) for 1 week, and pLenti-iCre-Neo-transfected immortalized cells were selected. Transfected immortalized cells were then subjected to the limiting dilution method to generate a monoclonal stable cell line.

### Statistical analysis

Statistical analysis was performed via GraphPad Prism 9.4.0 (GraphPad, La Jolla, CA, USA). To assess the normal distribution of the data, we used the D’Agostino‒Pearson normality test, Shapiro‒Wilk normality test or Anderson‒Darling test. After normality was analyzed, *P* values were calculated. All the statistical tests used in this study are described in the figure legends. The Kaplan‒Meier method was used for survival analysis. A *P* value < 0.05 was considered to indicate statistical significance. All of the data are expressed as the means ± SDs. All of the in vitro experiments were repeated at least three times unless otherwise indicated.

## Supplementary information

Supplementary Figures

Supplementary Table 1

Original western blots

## Data Availability

All data will be made available immediately after publication on request. Supplementary information is available at Cell Death and Differentiation’s website.
