# Supplementary material for: Collaborative orchestration of BH3-only proteins governs Bak/Bax-dependent hepatocyte apoptosis under antiapoptotic protein-deficiency in mice
Source: Cell Death Differ. 2025 Feb 24;32(6):1153–66. doi: 10.1038/s41418-025-01458-y (PMC12162870; doi:10.1038/s41418-025-01458-y)
Supplement: Supplementary file 1 — Supplementary Figures [file 41418_2025_1458_MOESM1_ESM.pdf]

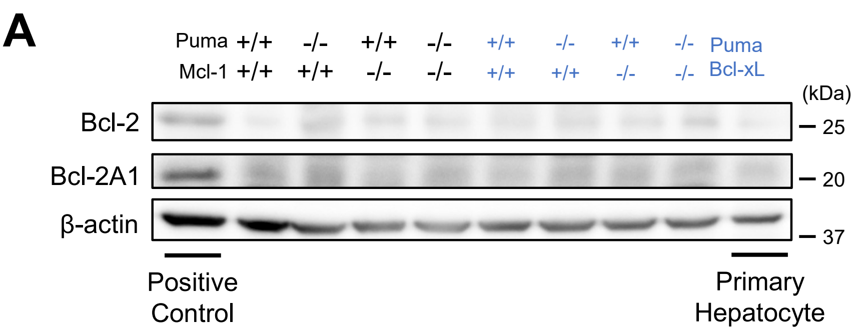

Supplementary Figure 1. Bcl-2 and Bcl-2A1 are nearly undetectable in *Mcl-1$^{flox/flox}$ Alb-Cre Puma$^{-/-}$* mice, *Bcl-xL$^{flox/flox}$ Alb-Cre Puma$^{-/-}$* mice and control mice.

(A) Western blots of Bcl-2, Bcl-2A1 and β-actin. The cell lysate of Hep-55.1 C cell line was used as a positive control. Lysate from wild-type C57BL/6J murine primary hepatocytes was used as “Primary Hepatocyte”.

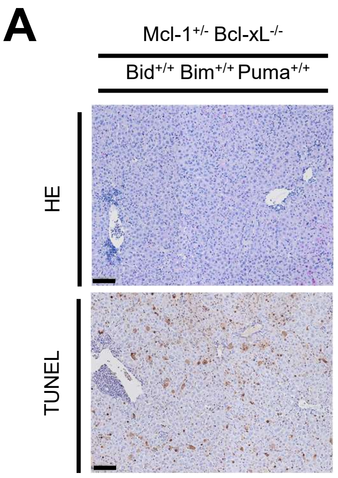

Supplementary Figure 2. ***Bcl-xL$^{flox/flox}$ Mcl-1$^{flox/+}$ Alb-Cre*** **mice exhibit severe hepatocyte apoptosis.** (**A**) Representative images of H&E staining and TUNEL staining of *Bcl-xL$^{flox/flox}$ Mcl-1$^{flox/+}$ Alb-Cre* mice. Scale bar (**A**): 100 µm

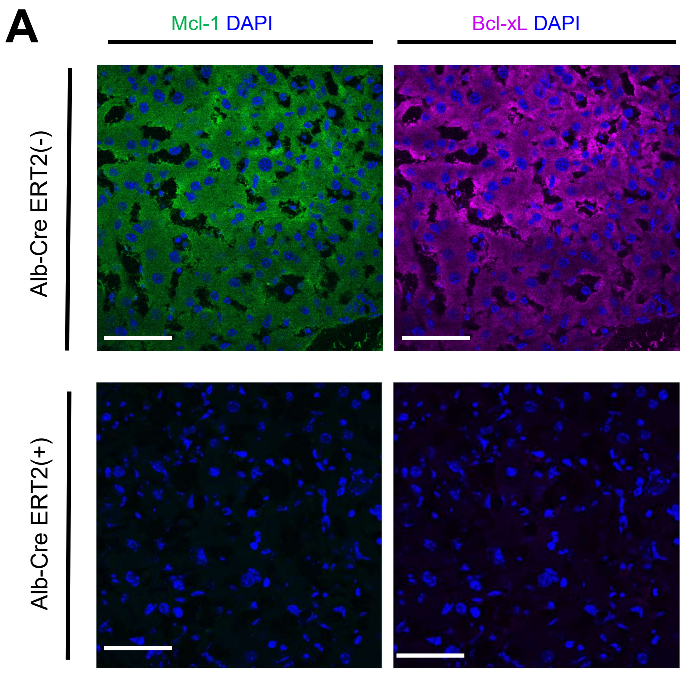

Supplementary Figure 3. Tamoxifen injection reduces the expression of Bcl-xL and Mcl-1.
Bcl-xL$^{flox/flox}$ *Mcl-1*$^{flox/flox}$ *Alb-Cre*^{ERT2} *Bid*$^{-/-}$ *Bim*$^{-/-}$ *Puma*$^{-/-}$ mice and control (*Bcl-xL*$^{flox/flox}$ *Mcl-1*$^{flox/flox}$ *Bid*$^{-/-}$ *Bim*$^{-/-}$ *Puma*$^{-/-}$) mice were injected intraperitoneally with 1 mg of tamoxifen for 3 consecutive days and analyzed 24 hours after the last tamoxifen injection. (**A**) Immunofluorescence staining for Mcl-1 and Bcl-xL. Scale bar (**A**): 50 μm

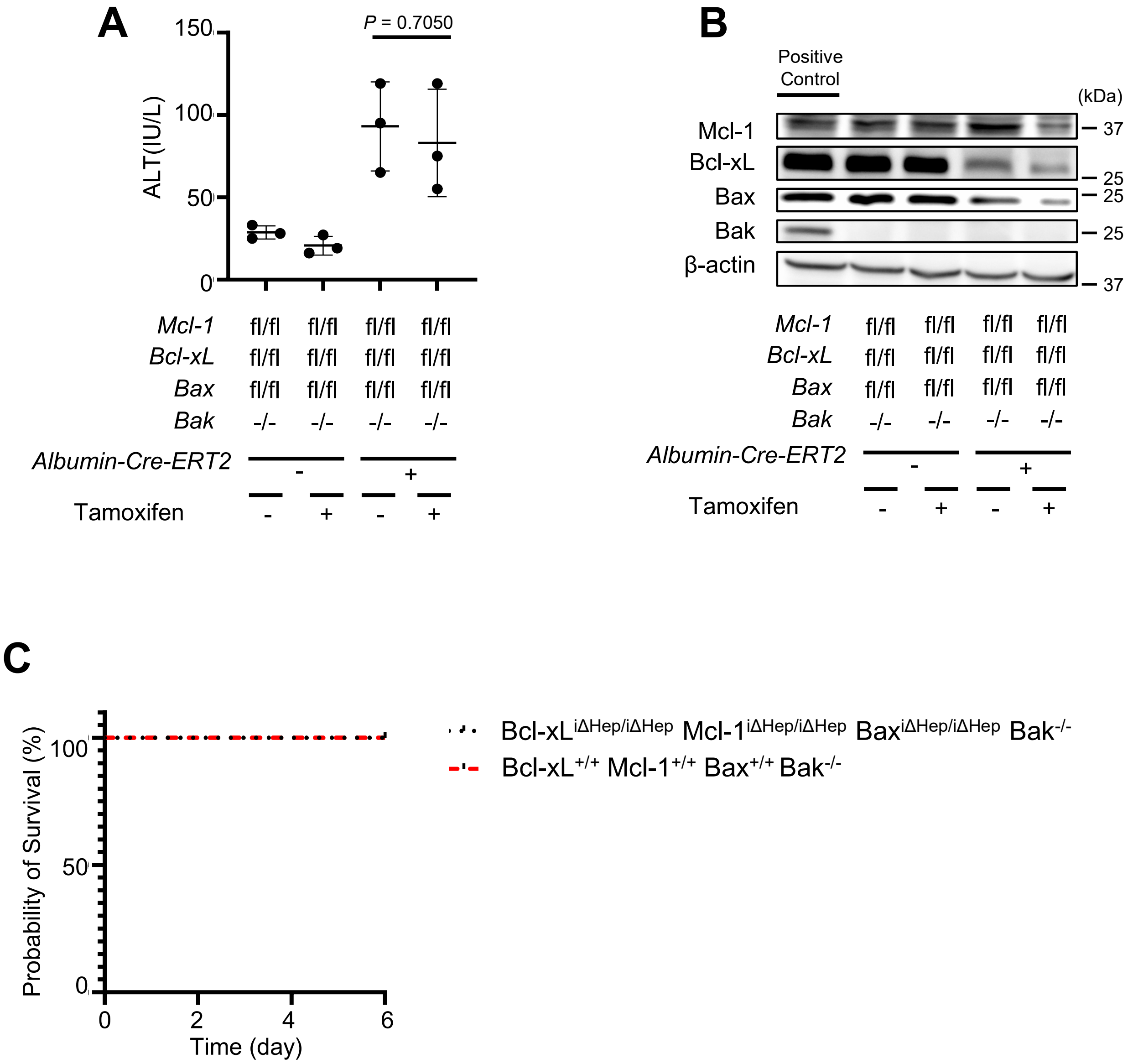

Supplementary Figure 4. Bax/Bak deficiency blocks the hepatocyte apoptosis induced by Mcl-1/Bcl-xL deficiency.

(**A-C**) *Bcl-xL*$^{flox/flox}$ *Mcl-1*$^{flox/flox}$ *Bax*$^{flox/flox}$ *Alb-Cre ERT2 Bak*$^{-/-}$ mice and control (*Bcl-xL*$^{flox/flox}$ *Mcl-1*$^{flox/flox}$ *Bax*$^{flox/flox}$ *Bak*$^{-/-}$) mice were injected intraperitoneally with 1 mg of tamoxifen for 5 consecutive days and analyzed at day 6; n=3 per group. (**A**) Serum ALT levels before and after tamoxifen injection. (**B**) Western blot analysis before and after tamoxifen injection. (**C**) Probability of survival after tamoxifen injection. Data was analyzed by two-tailed unpaired *t*-test (**A**) or the log-rank test (**C**).

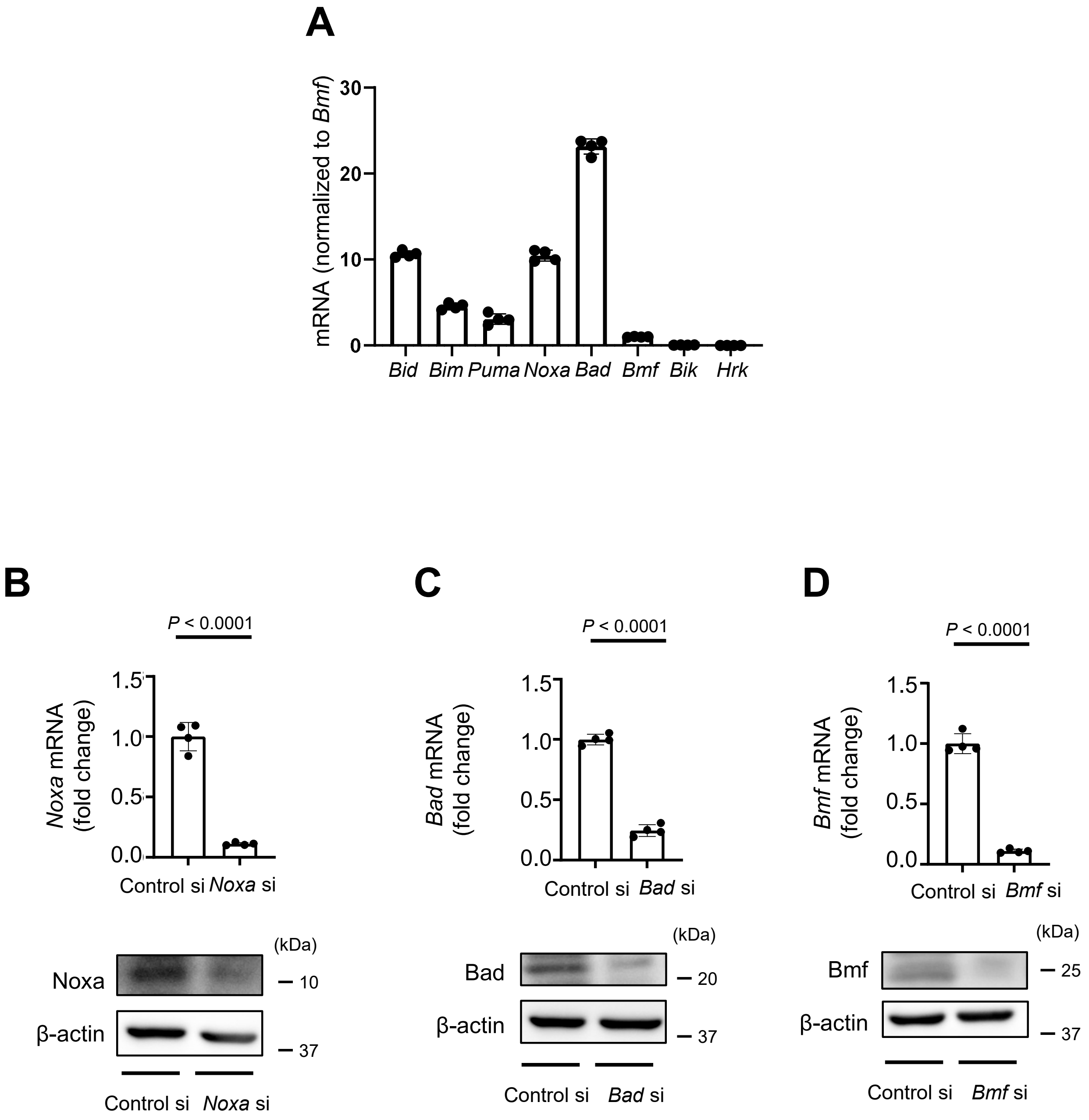

Supplementary Figure 5. Knockdown of Noxa suppresses apoptosis in Bcl-xL-, Mcl-1-, Bid-, Bim- and Puma-deficient hepatocytes.

(**A**) Relative mRNA expression levels of all BH3-only proteins in the wild-type C57BL/6J murine primary hepatocytes. Expression levels of each protein were normalized to *Bmf* mRNA level. (**B-D**) Relative mRNA expression levels and Western blots of (**B**) *Noxa*, (**C**) *Bad* and (**D**) *Bmf* after siRNA transfection. Data is represented as mean ± SD. Data was analyzed by two-tailed unpaired *t*-tests (**B-D**).

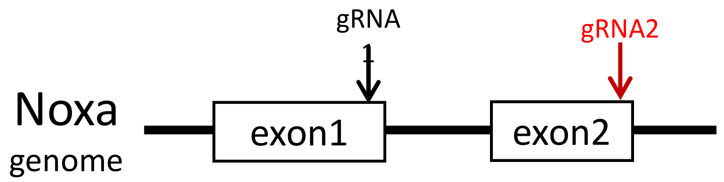

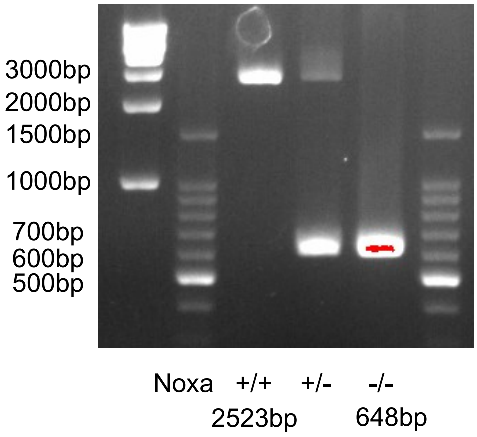

Supplementary Figure 6. Generation of Noxa knockout mice by CRISPR-Cas9 technology.
(**A**) Schematic of Noxa knockout in mice using CRISPR-Cas9 system. The gRNA sequences were designed using the online tools http://crispor.tefor.net/ and https://crispr.dbcls.jp/ to predict unique target sites throughout the mouse genome (gRNA1: 5'- ACGCGCCAGTGAACCCAACG -3' and gRNA2: 5'-CTGGGAAGTCGCAAAAGAGC -3'). Specific Alt-R CRISPR-Cas9 crRNA was procured from IDT and assembled with Alt-R CRISPR-Cas9 tracrRNA, following the manufacturer's instructions. (**B**) Geno typing band of Noxa knockout mice. $\textit{Noxa}^{+/+}$: 2523 bp, $\textit{Noxa}^{+/-}$: 2523 bp + 648 bp, $\textit{Noxa}^{-/-}$: 648 bp.

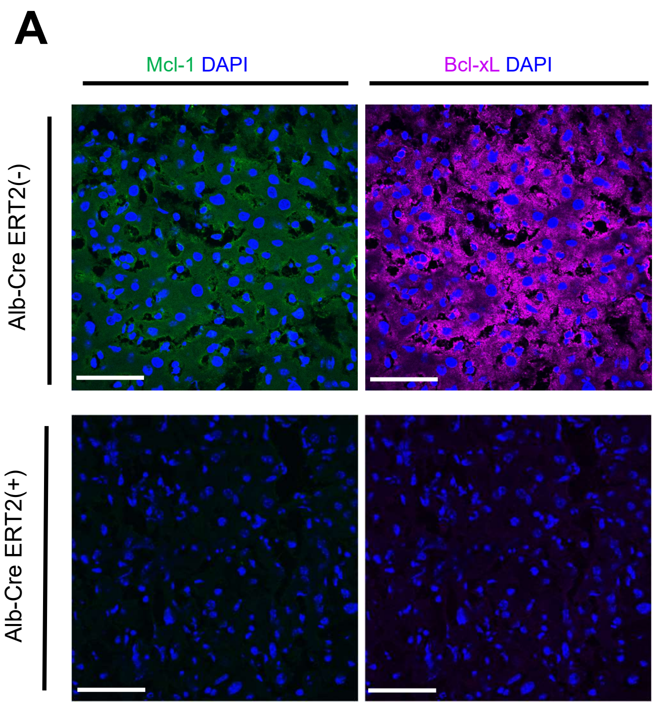

Supplementary Figure 7. Bcl-xL and Mcl-1 are decreased after tamoxifen injection.
Bcl-xL$^{flox/flox}$ Mcl-1$^{flox/flox}$ Alb-Cre ERT2 Bid$^{-/-}$ Bim$^{-/-}$ Puma$^{-/-}$ Noxa$^{-/-}$ mice and control (*Bcl-xL$^{flox/flox}$ Mcl-1$^{flox/flox}$ Bid$^{-/-}$ Bim$^{-/-}$ Puma$^{-/-}$ Noxa$^{-/-}$*) mice were injected intraperitoneally with 1 mg of tamoxifen and sacrificed at 12 hours after tamoxifen injection. (**A**) Immunofluorescence staining for Mcl-1 and Bcl-xL. Scale bar (**A**): 50 μm

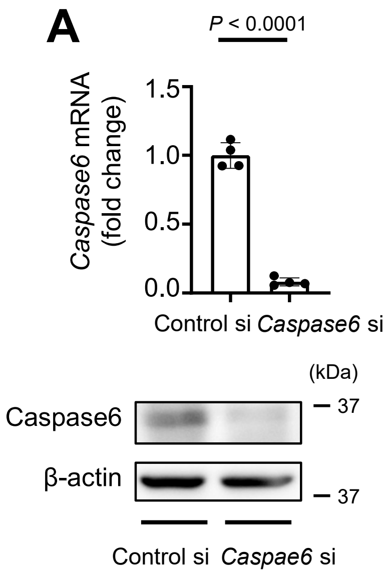

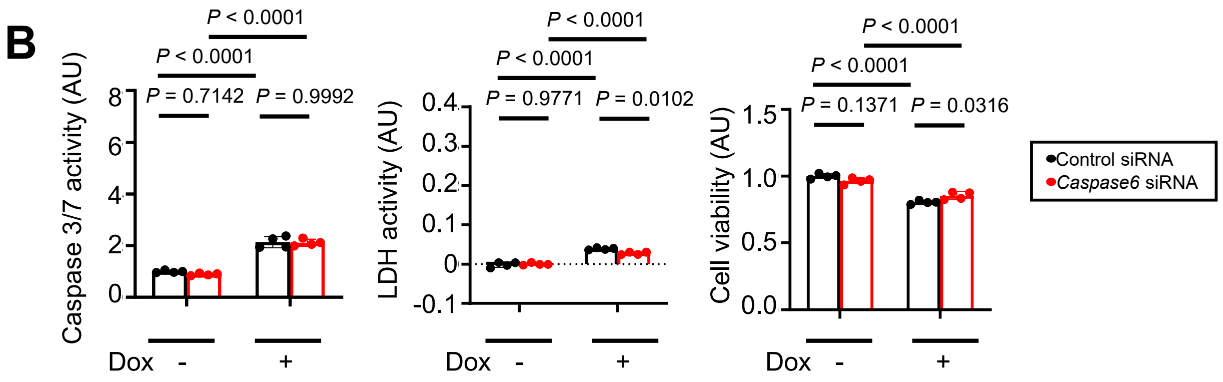

Supplementary Figure 8. Knockdown of Caspase-6 increases relative cell viability in Bcl-xL-, Mcl-1-, Bid-, Bim-, Puma-, and Noxa-deficient hepatocytes.
Immortalized *Bcl-xL*$^{flox/flox}$ *Mcl-1*$^{flox/flox}$ *Bid*$^{-/-}$ *Bim*$^{-/-}$ *Puma*$^{-/-}$ *Noxa*$^{-/-}$ mouse primary hepatocytes with doxycycline-inducible Cre recombinase were treated with 0.3 μM doxycycline for 48 hours after transfection of *caspase-6* siRNA or control siRNA. (**A**) Relative mRNA expression levels and Western blotting of caspase-6 after siRNA transfection. (**B**) Caspase 3/7 activity and LDH activity in the culture supernatant, cell viability assessed with a WST assay after *caspase-6* knockdown. Data is represented as mean ± SD. Data was analyzed by two-tailed unpaired *t*-test (**A**) or One-way ANOVA with Sidak's multiple comparisons test (**B**).

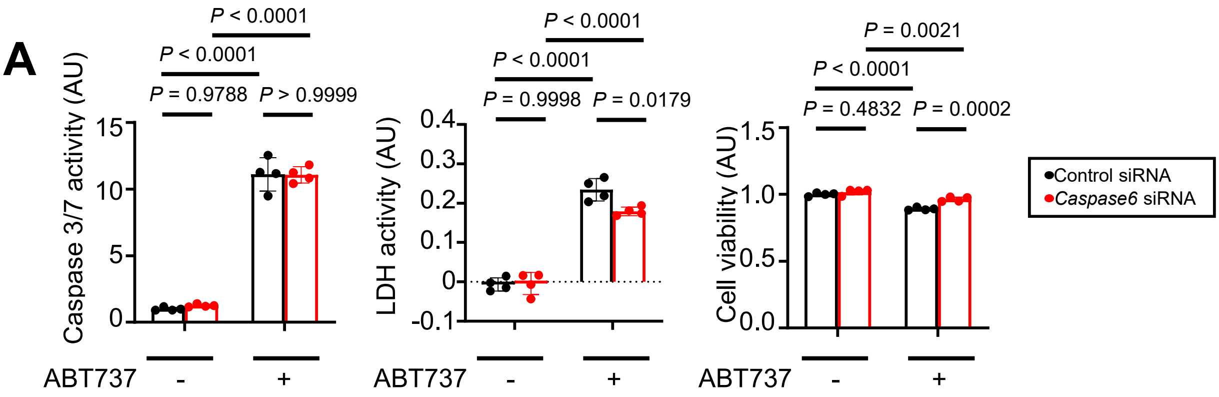

Supplementary Figure 9. Knockdown of Caspase-6 improves the relative cell viability of BNL.CL2. cells treated with ABT-737.

BNL.CL2. cells were treated with 4 µM ABT-737, which is the Bcl-xL inhibitor, for 24 hours after transfection of *caspase-6* siRNA or control siRNA. (**A**) Caspase 3/7 activity and LDH activity in the culture supernatant, cell viability assessed with a WST assay after *caspase-6* knockdown. Data is represented as mean ± SD. Data was analyzed by One-way ANOVA with Sidak's multiple comparisons test (**A**).
