# Supplementary material for: Collaborative orchestration of BH3-only proteins governs Bak/Bax-dependent hepatocyte apoptosis under antiapoptotic protein-deficiency in mice
Source: Cell Death Differ. 2025 Feb 24;32(6):1153–66. doi: 10.1038/s41418-025-01458-y (PMC12162870; doi:10.1038/s41418-025-01458-y)

Fig 1A

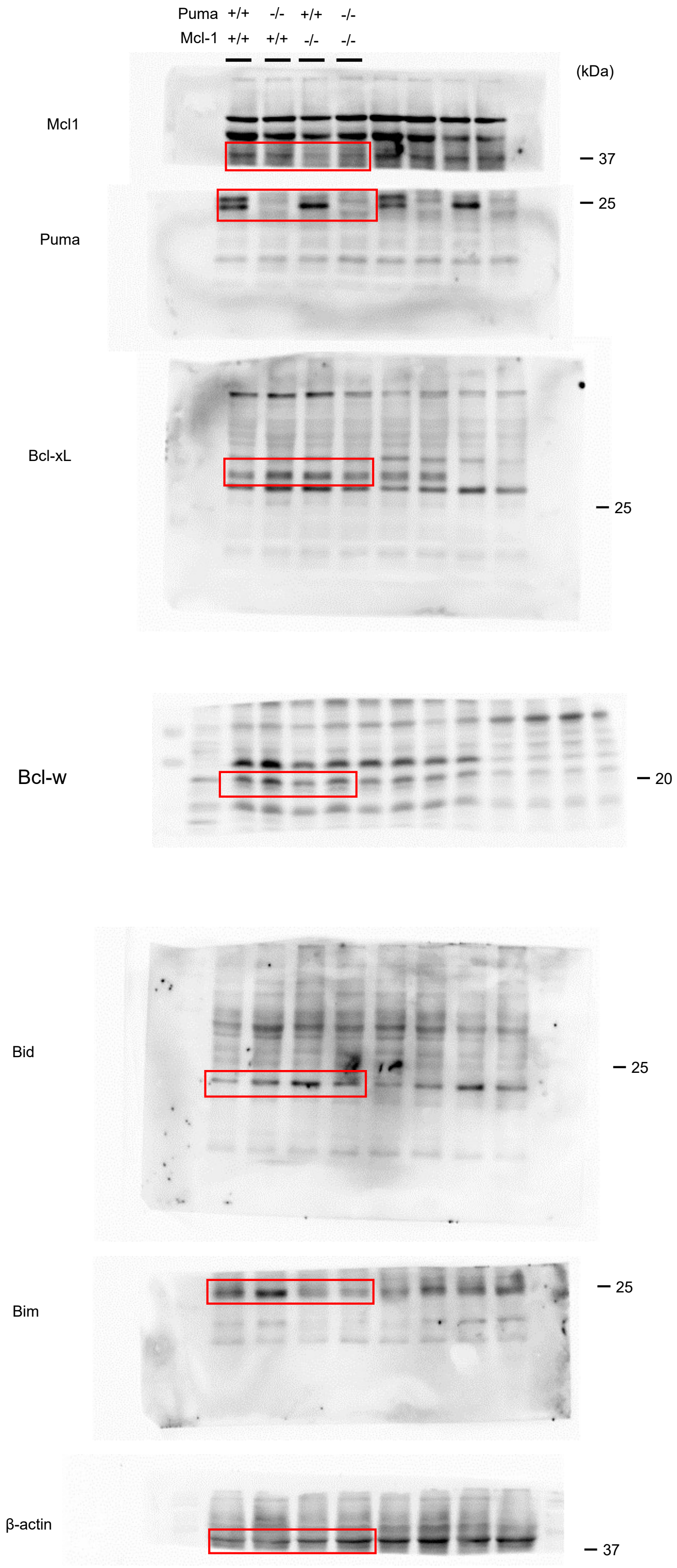
Puma +/+ -/- +/+ -/-
Mcl-1 +/+ +/+ -/- -/-
(kDa)
Mcl1
— 37
Puma
— 25
Bcl-xL
— 25
Bcl-w
— 20
Bid
— 25
Bim
— 25
β-actin
— 37

Fig 1B

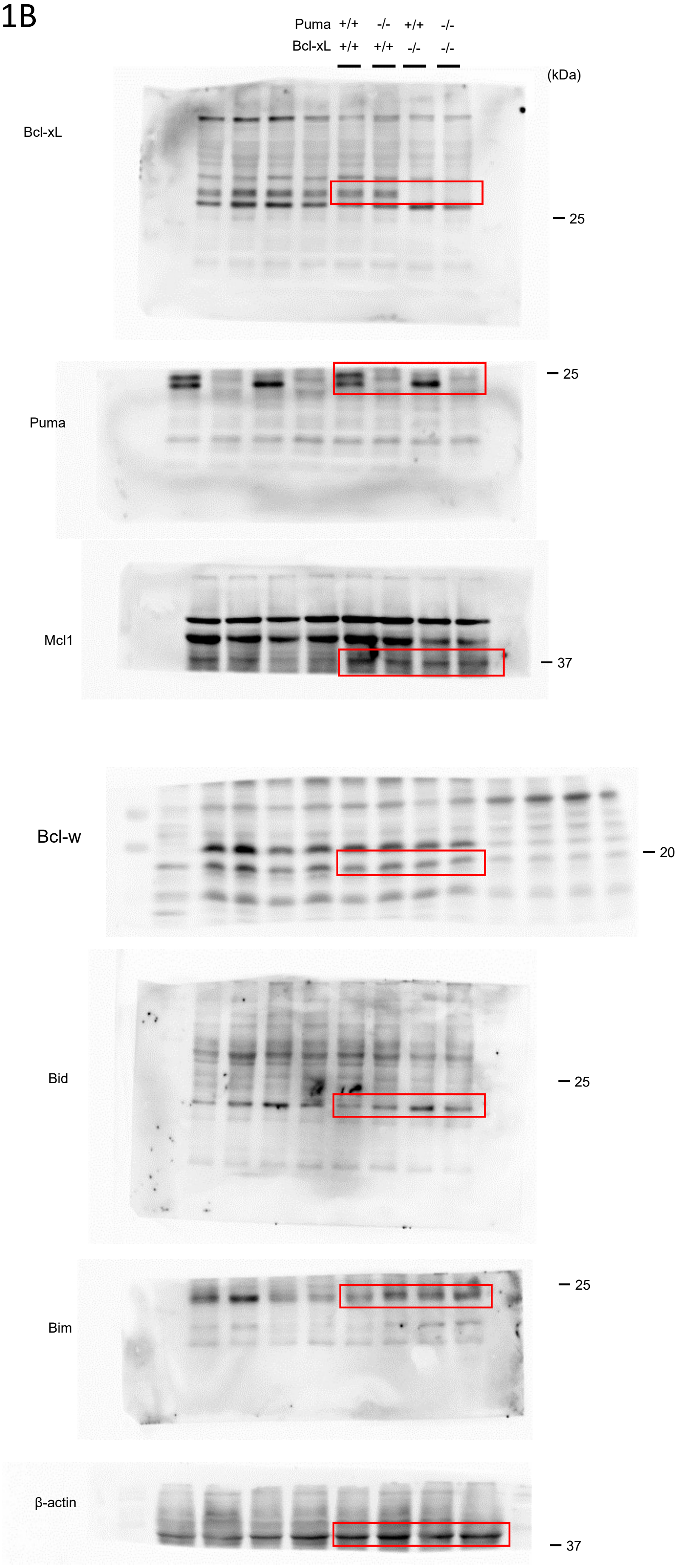

Puma +/+ -/- +/+ -/-
Bcl-xL +/+ +/+ -/- -/-
(kDa)
Bcl-xL
25
Puma
25
Mcl1
37
Bcl-w
20
Bid
25
Bim
25
β-actin
37

Fig 1G

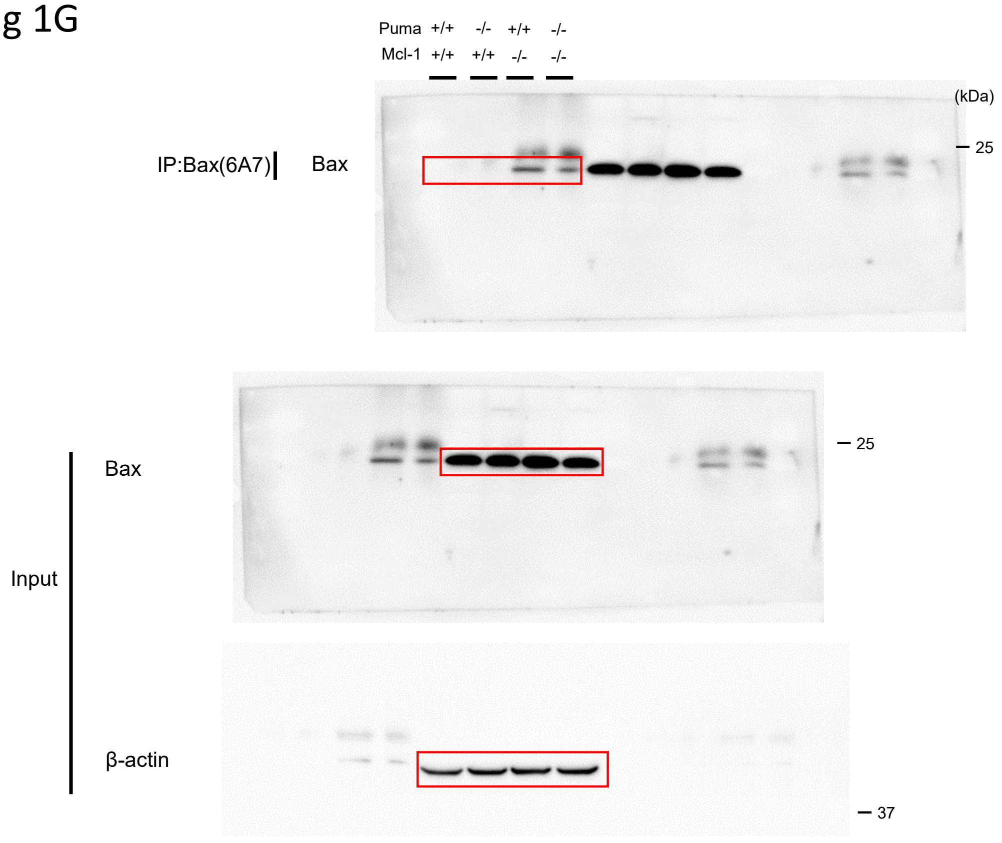

Puma +/+ -/- +/+ -/-
Mcl-1 +/+ +/+ -/- -/-
(kDa)
25
IP:Bax(6A7)
Bax
Bax
25
Input
β-actin
37

Fig 1H

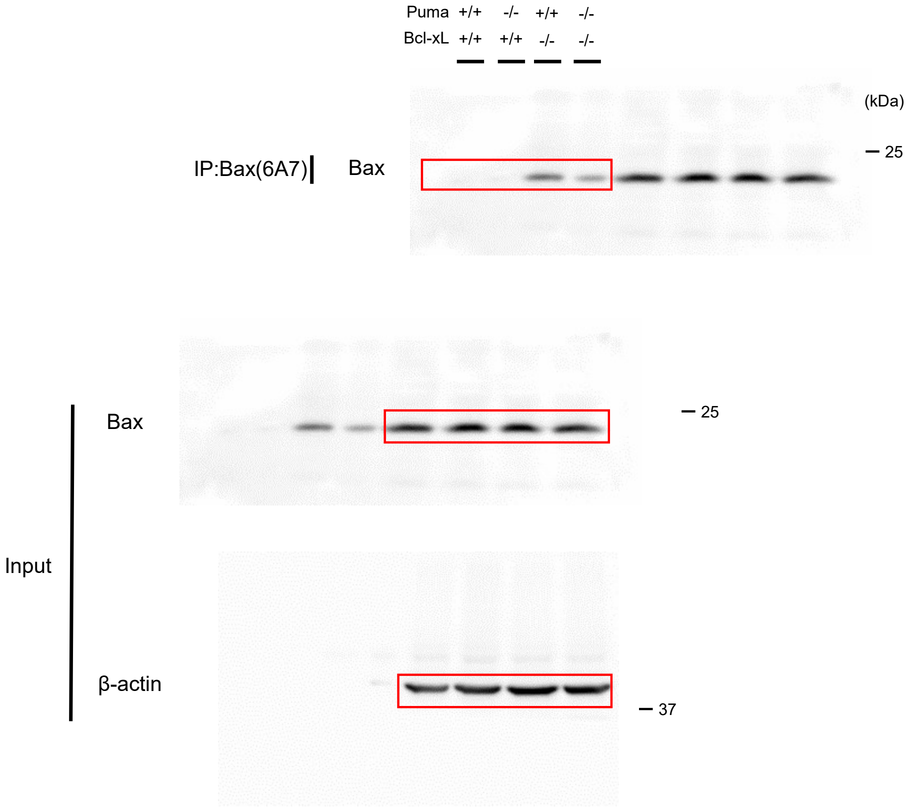

Puma +/+ -/- +/+ -/-
Bcl-xL +/+ +/+ -/- -/-
(kDa)
25
IP:Bax(6A7)
Bax
Bax
25
Input
β-actin
37

Fig 2A

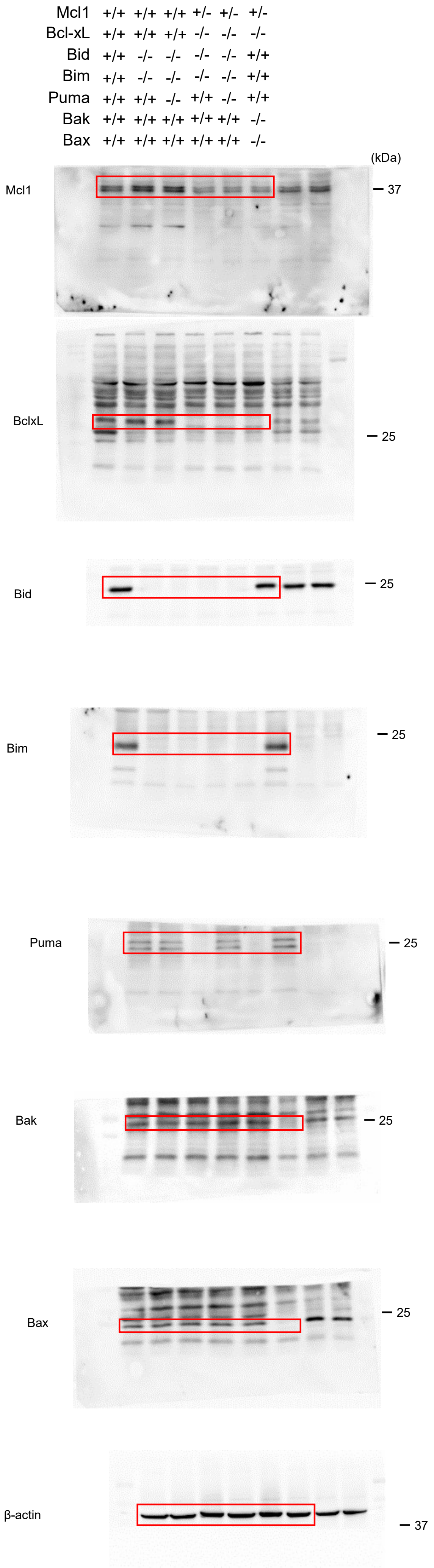

Mcl1 +/+ +/+ +/+ +/- +/- +/-
Bcl-xL +/+ +/+ +/+ -/- -/- -/-
Bid +/+ -/- -/- -/- -/- +/+
Bim +/+ -/- -/- -/- -/- +/+
Puma +/+ +/+ -/- +/+ -/- +/+
Bak +/+ +/+ +/+ +/+ +/+ -/-
Bax +/+ +/+ +/+ +/+ +/+ -/-
(kDa)
Mcl1
37
BclxL
25
Bid
25
Bim
25
Puma
25
Bak
25
Bax
25
β-actin
37

Fig 2E

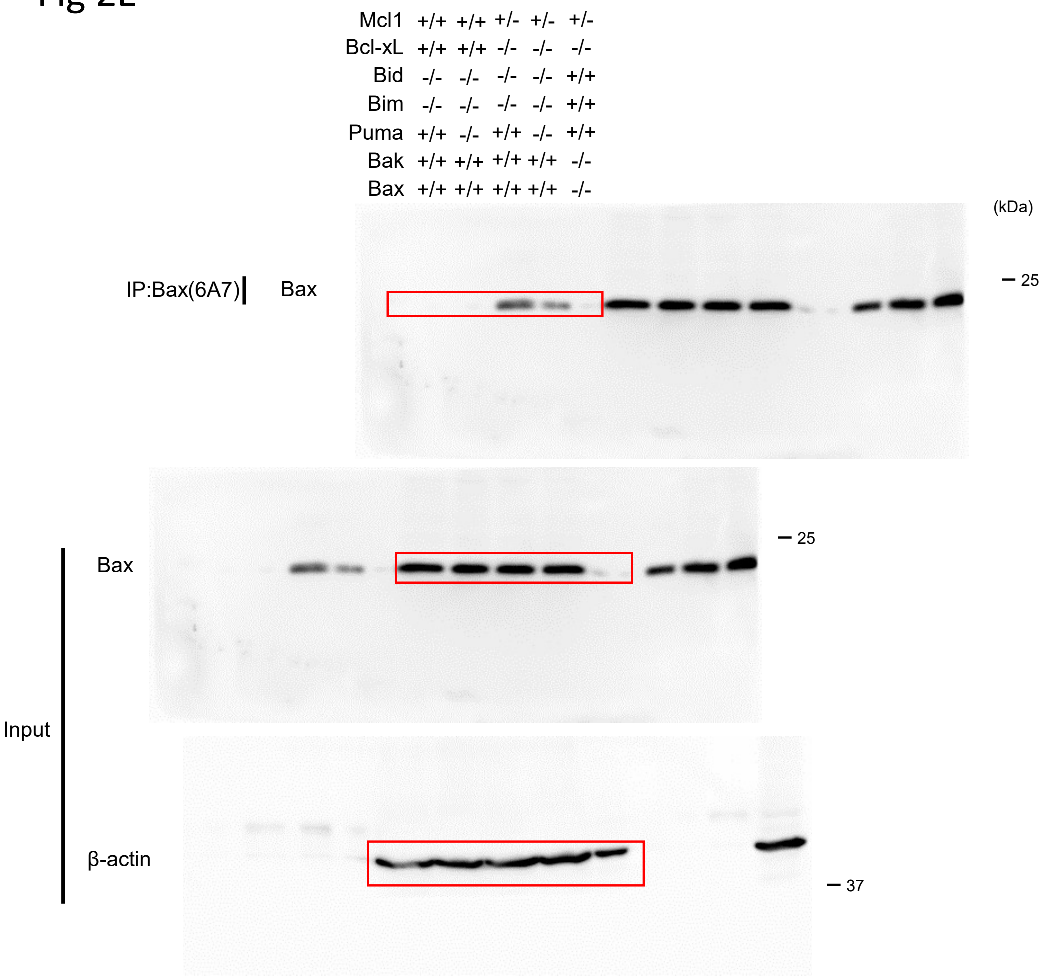

Mcl1 +/+ +/+ +/- +/- +/-
Bcl-xL +/+ +/+ -/- -/- -/-
Bid -/- -/- -/- -/- +/+
Bim -/- -/- -/- -/- +/+
Puma +/+ -/- +/+ -/- +/+
Bak +/+ +/+ +/+ +/+ -/-
Bax +/+ +/+ +/+ +/+ -/-
(kDa)
IP:Bax(6A7)
Bax
25
Bax
25
Input
β-actin
37

Fig 2F

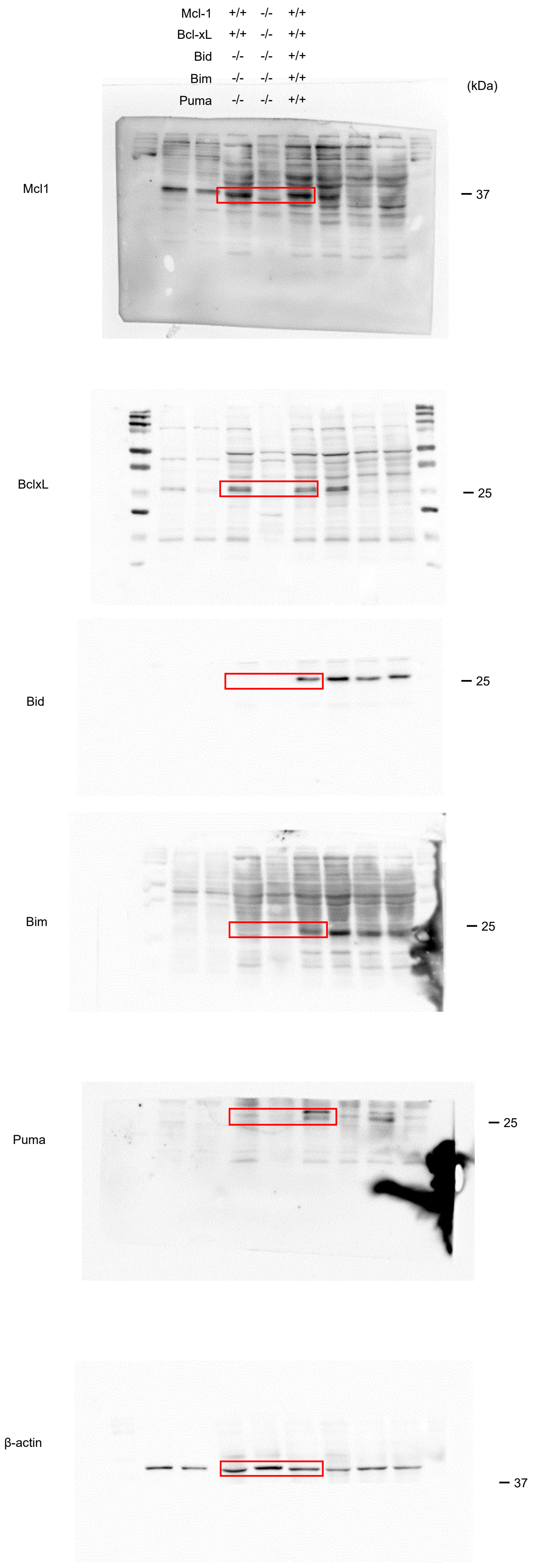

Mcl-1 +/+ -/- +/+
Bcl-xL +/+ -/- +/+
Bid -/- -/- +/+
Bim -/- -/- +/+
Puma -/- -/- +/+
(kDa)
Mcl1
37
BclxL
25
Bid
25
Bim
25
Puma
25
β-actin
37

Fig 2J

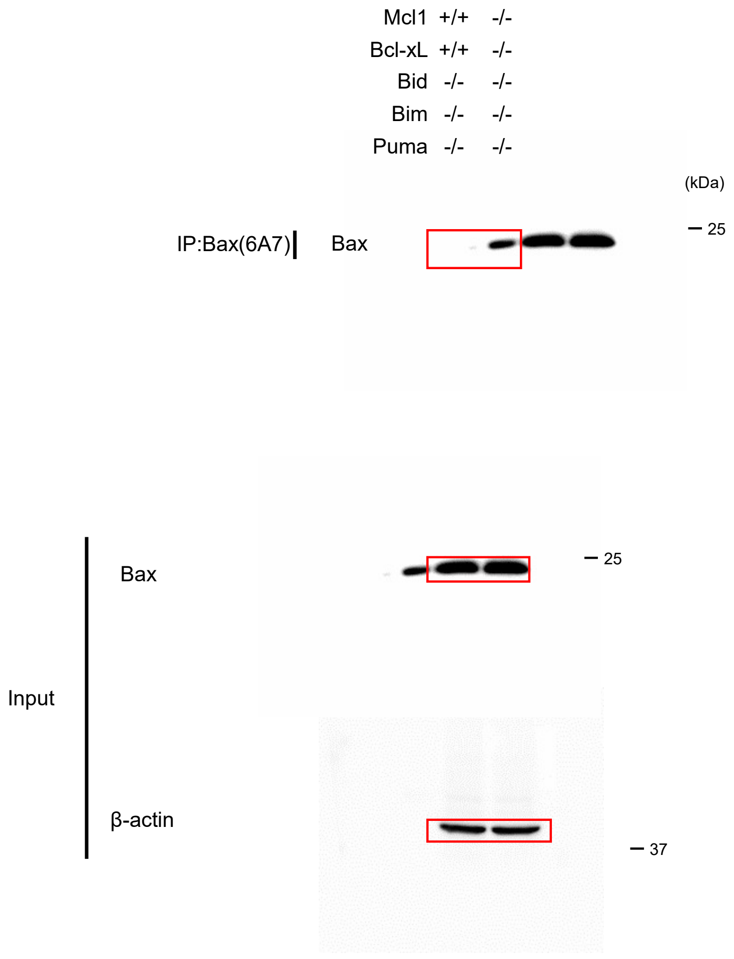

Mcl1 +/+ -/-
Bcl-xL +/+ -/-
Bid -/- -/-
Bim -/- -/-
Puma -/- -/-
(kDa)
IP:Bax(6A7)
Bax
25
Input
Bax
25
β-actin
37

Fig 3A

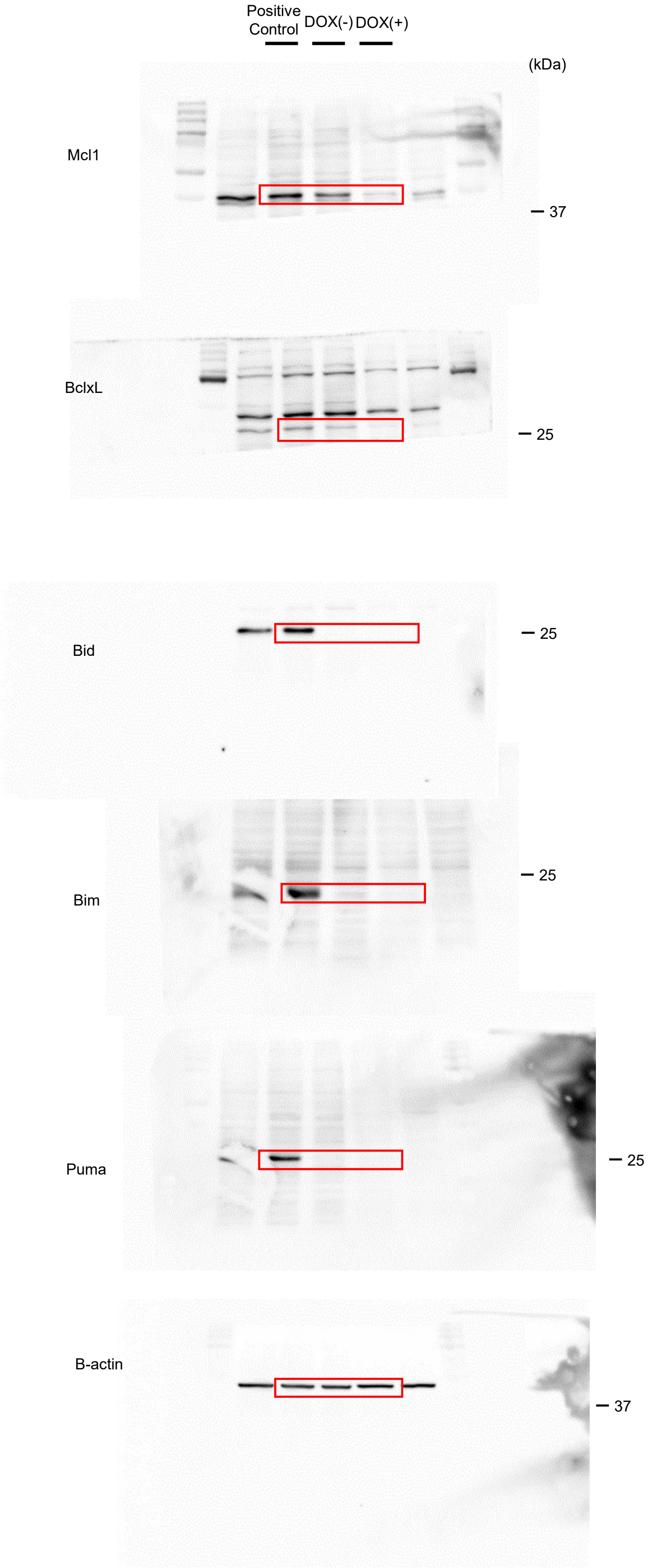
Positive Control
DOX(-)
DOX(+)
(kDa)
Mcl1
37
BclxL
25
Bid
25
Bim
25
Puma
25
B-actin
37

Fig 3C

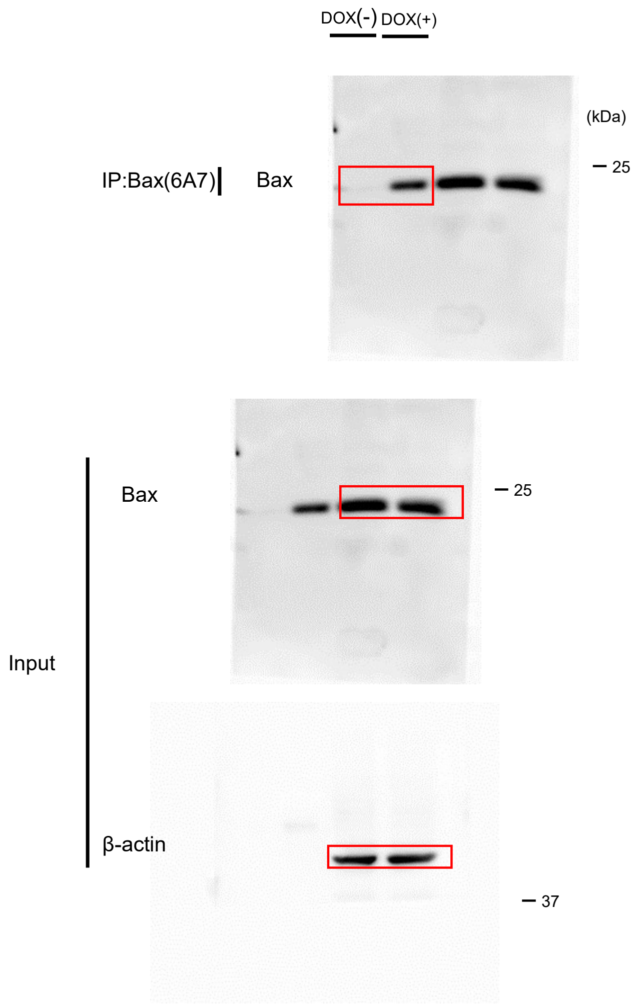

Fig 3E

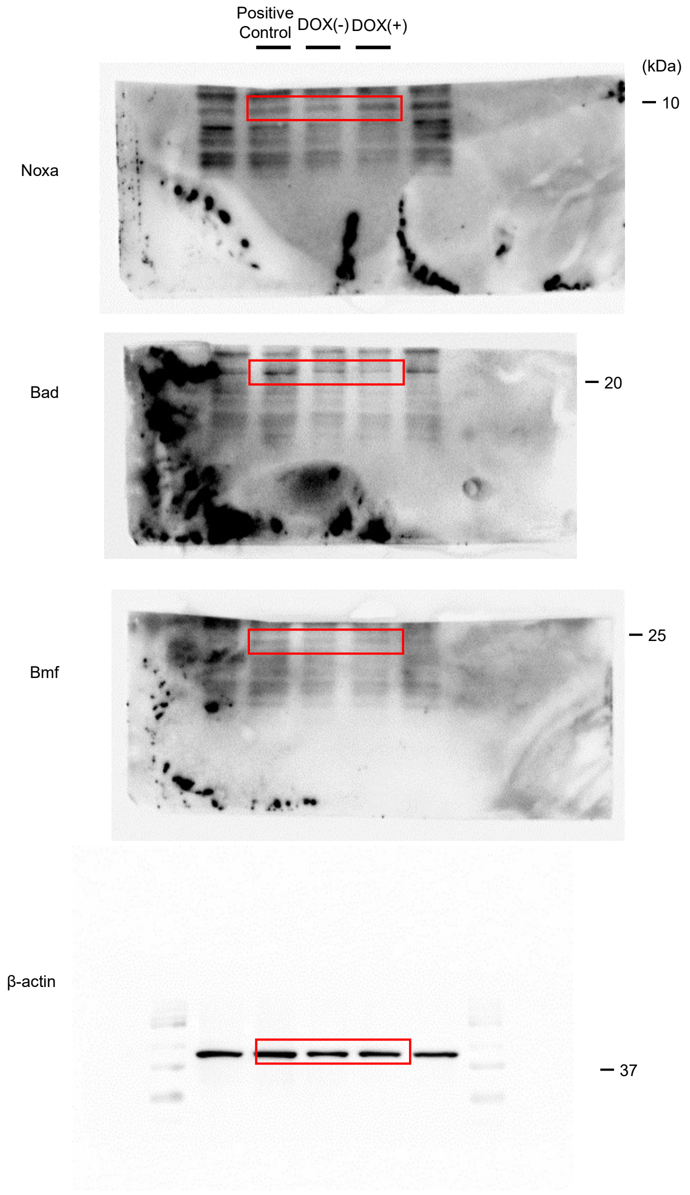

Fig 3F

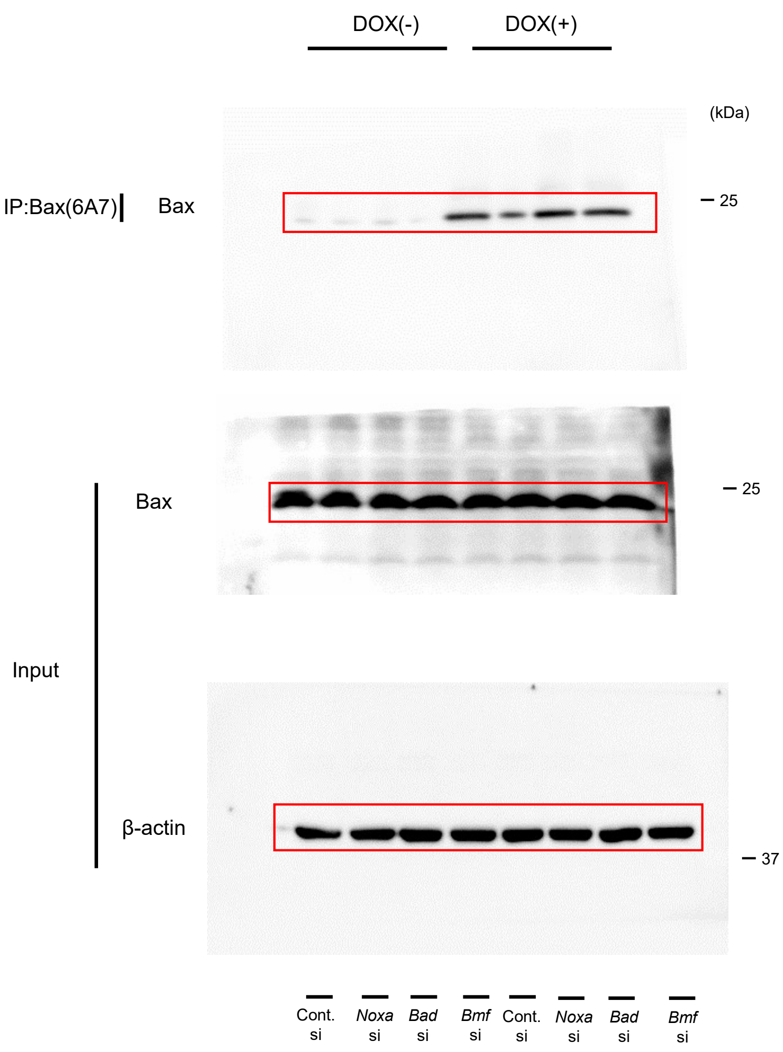
DOX(-)
DOX(+)
(kDa)
IP:Bax(6A7)
Bax
25
Input
Bax
25
β-actin
37
Cont. si
Noxa si
Bad si
Bmf si
Cont. si
Noxa si
Bad si
Bmf si

Fig 4A

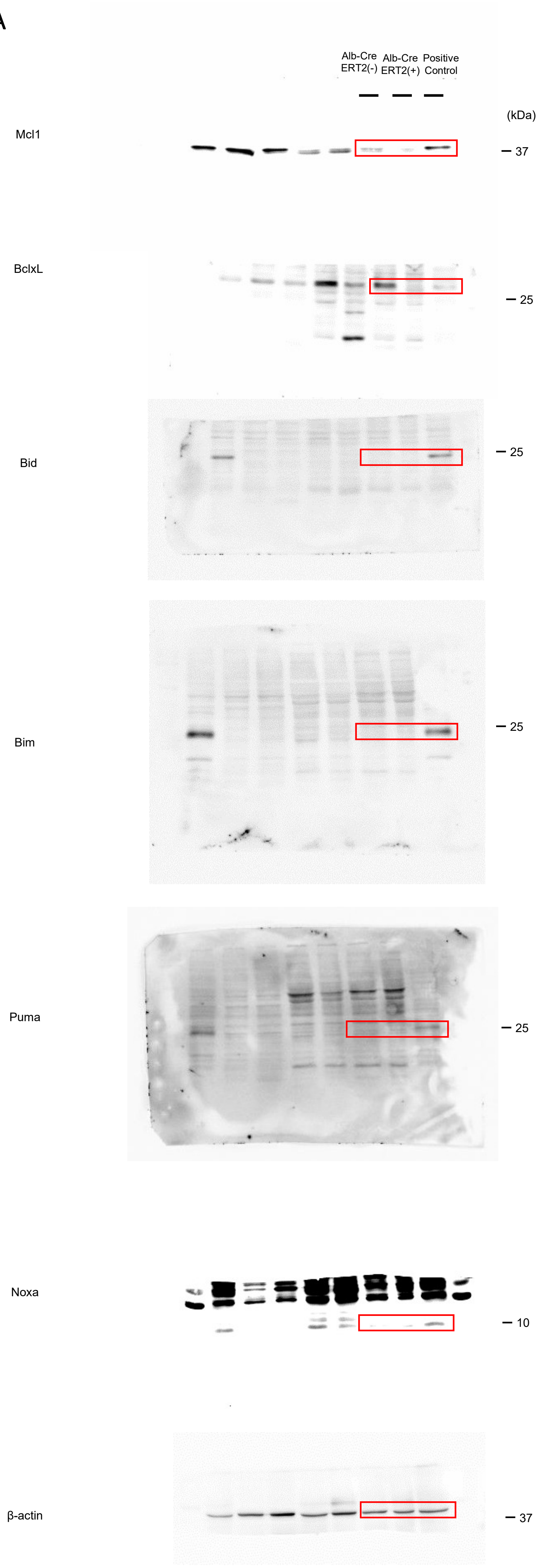

Alb-Cre ERT2(-)
Alb-Cre ERT2(+)
Positive Control
(kDa)
Mcl1
37
BclxL
25
Bid
25
Bim
25
Puma
25
Noxa
10
β-actin
37

Fig 4E

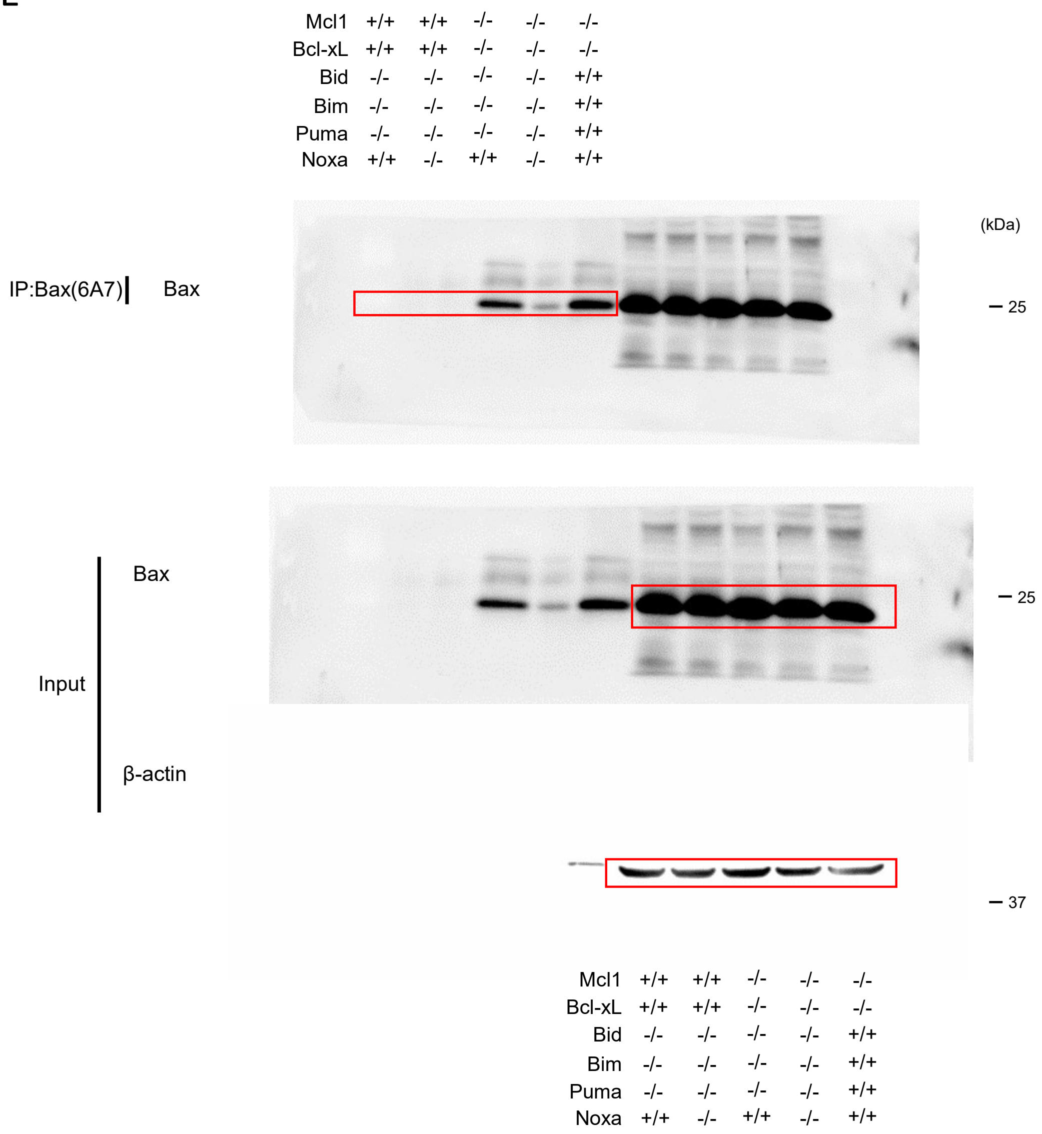

Mcl1 +/+ +/+ -/- -/- -/-
Bcl-xL +/+ +/+ -/- -/- -/-
Bid -/- -/- -/- -/- +/+
Bim -/- -/- -/- -/- +/+
Puma -/- -/- -/- -/- +/+
Noxa +/+ -/- +/+ -/- +/+
IP:Bax(6A7)
Bax
(kDa)
25
Input
Bax
β-actin
25
37
Mcl1 +/+ +/+ -/- -/- -/-
Bcl-xL +/+ +/+ -/- -/- -/-
Bid -/- -/- -/- -/- +/+
Bim -/- -/- -/- -/- +/+
Puma -/- -/- -/- -/- +/+
Noxa +/+ -/- +/+ -/- +/+

Fig 5A

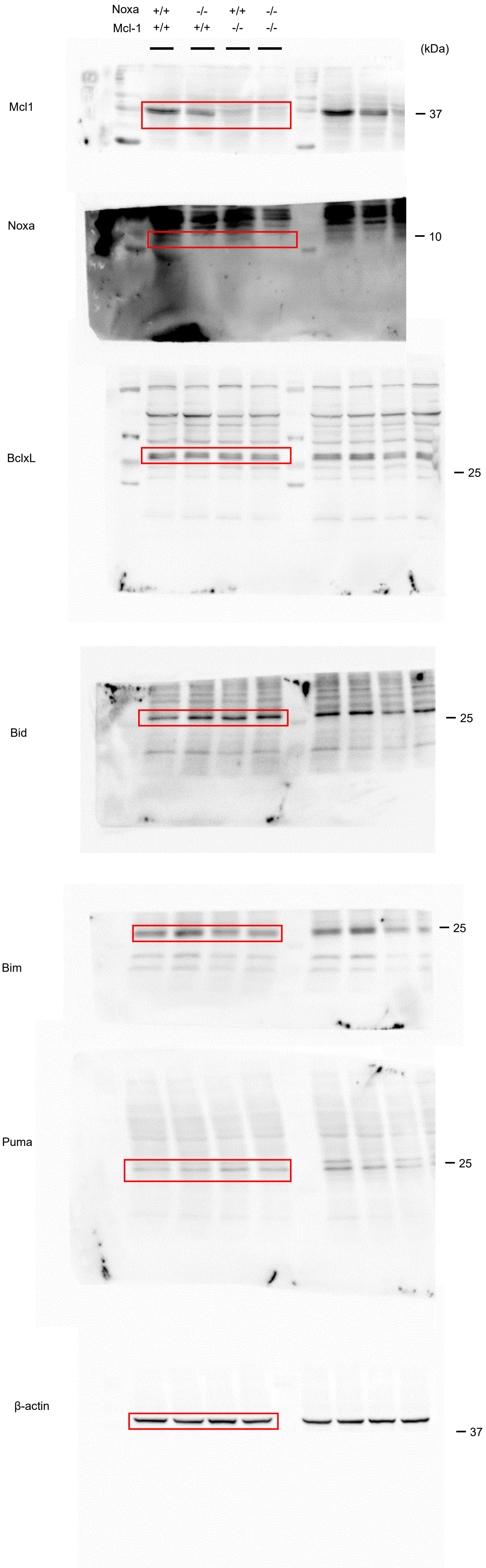
Noxa +/+ -/- +/+ -/-
Mcl-1 +/+ +/+ -/- -/-
(kDa)
Mcl1
37
Noxa
10
BclxL
25
Bid
25
Bim
25
Puma
25
β-actin
37

Fig 5E

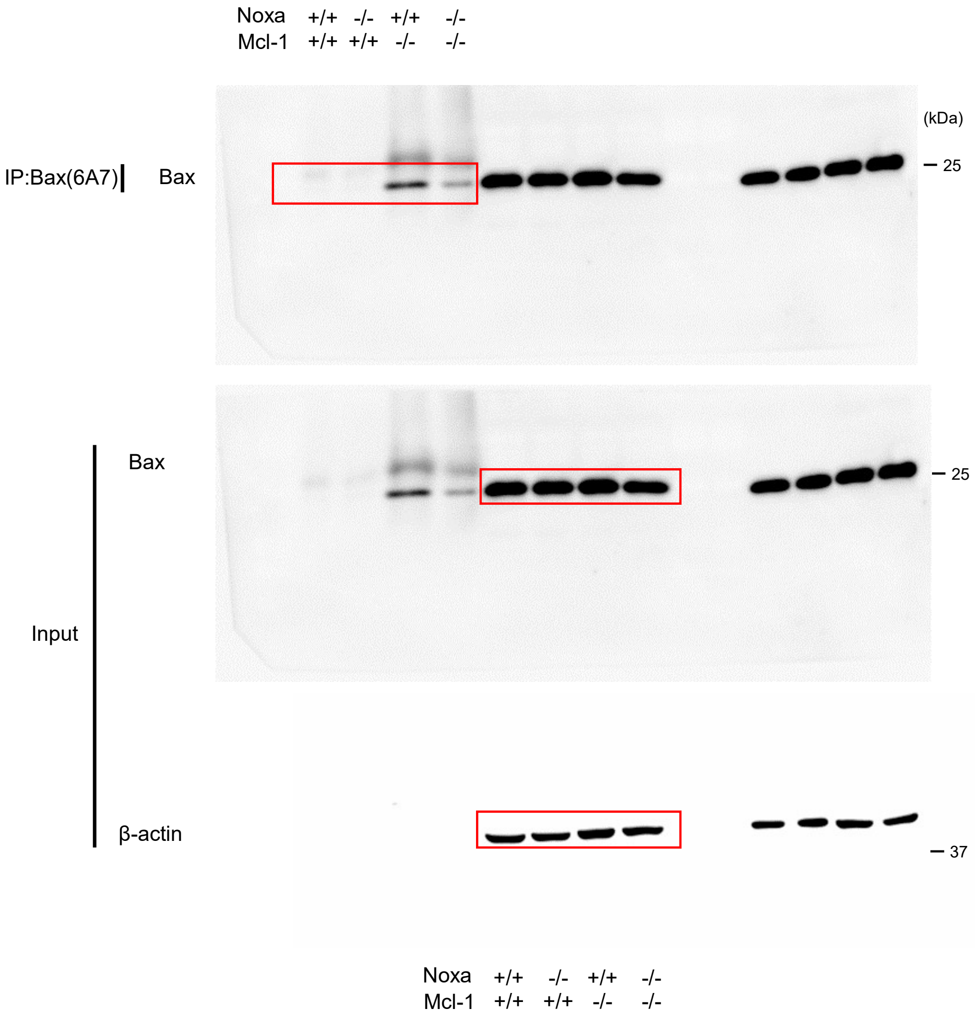

Noxa +/+ -/- +/+ -/-
Mcl-1 +/+ +/+ -/- -/-
(kDa)
IP:Bax(6A7)
Bax
25
Bax
25
Input
β-actin
37
Noxa +/+ -/- +/+ -/-
Mcl-1 +/+ +/+ -/- -/-

Fig 6A

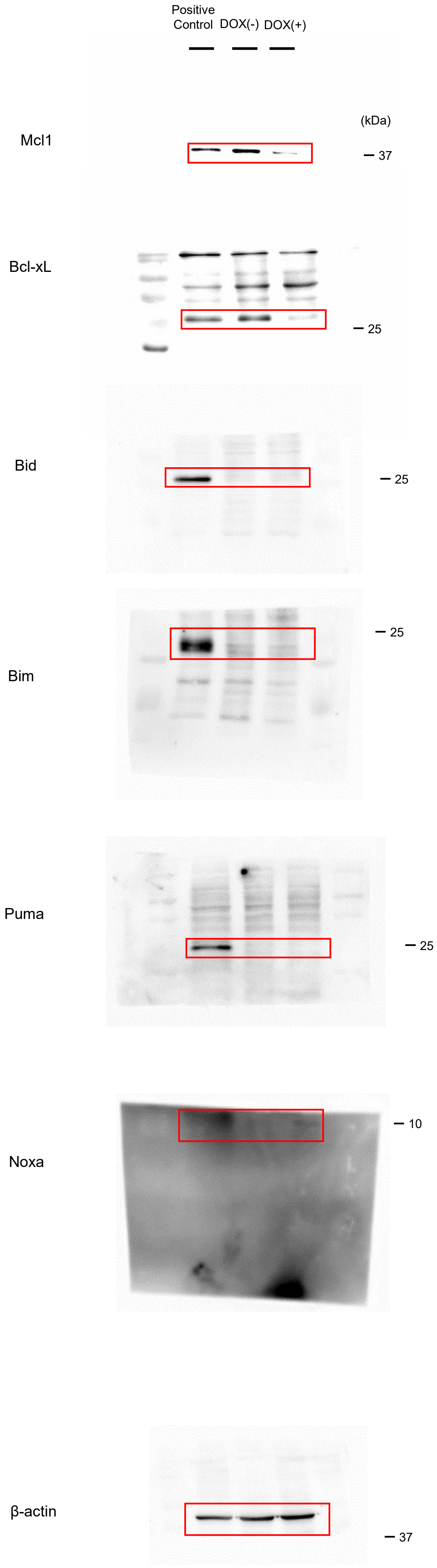

Positive Control
DOX(-)
DOX(+)
(kDa)
Mcl1
37
Bcl-xL
25
Bid
25
Bim
25
Puma
25
Noxa
10
β-actin
37

Fig 6C

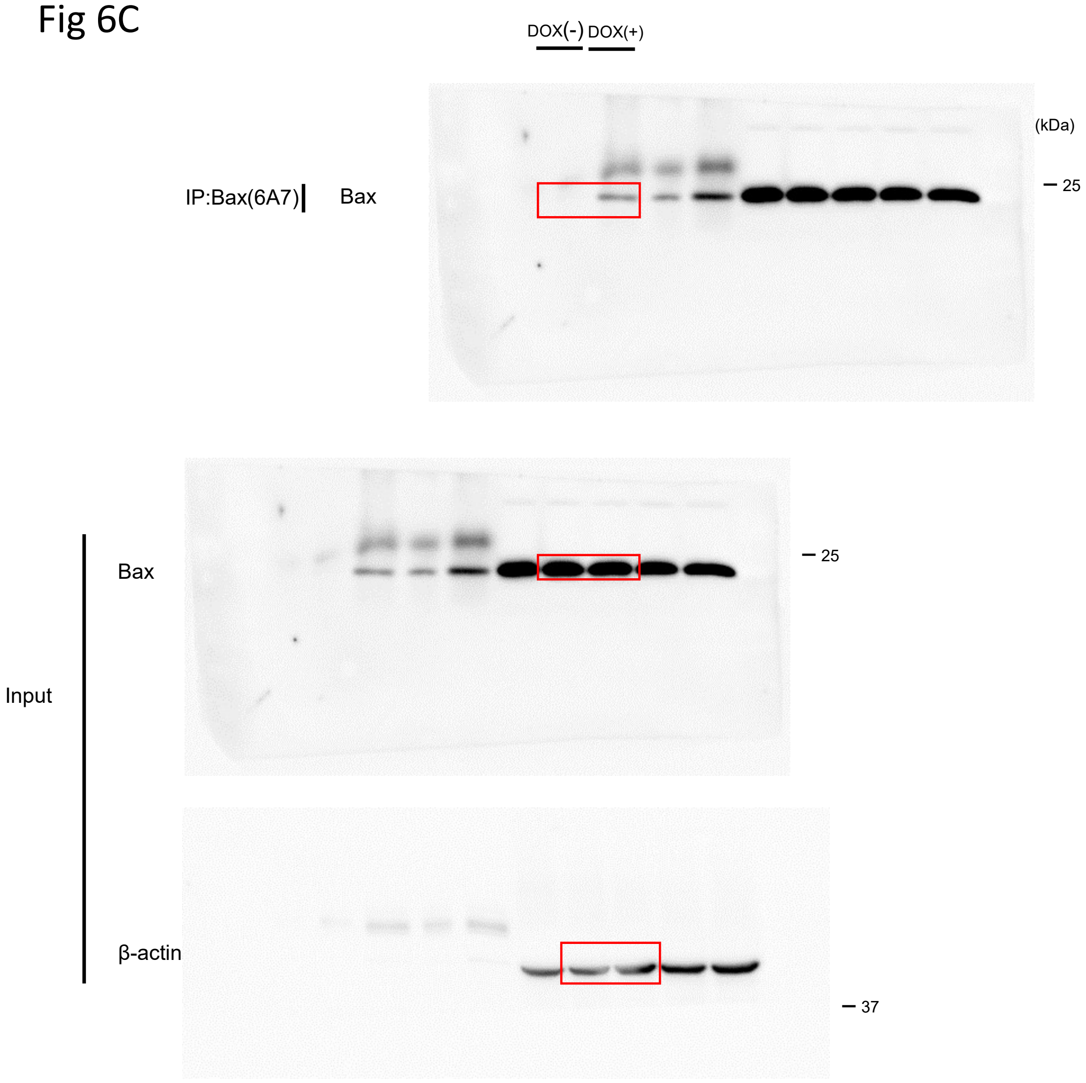

DOX(-)
DOX(+)
IP:Bax(6A7)
Bax
(kDa)
25
Input
Bax
25
β-actin
37

Fig 6E

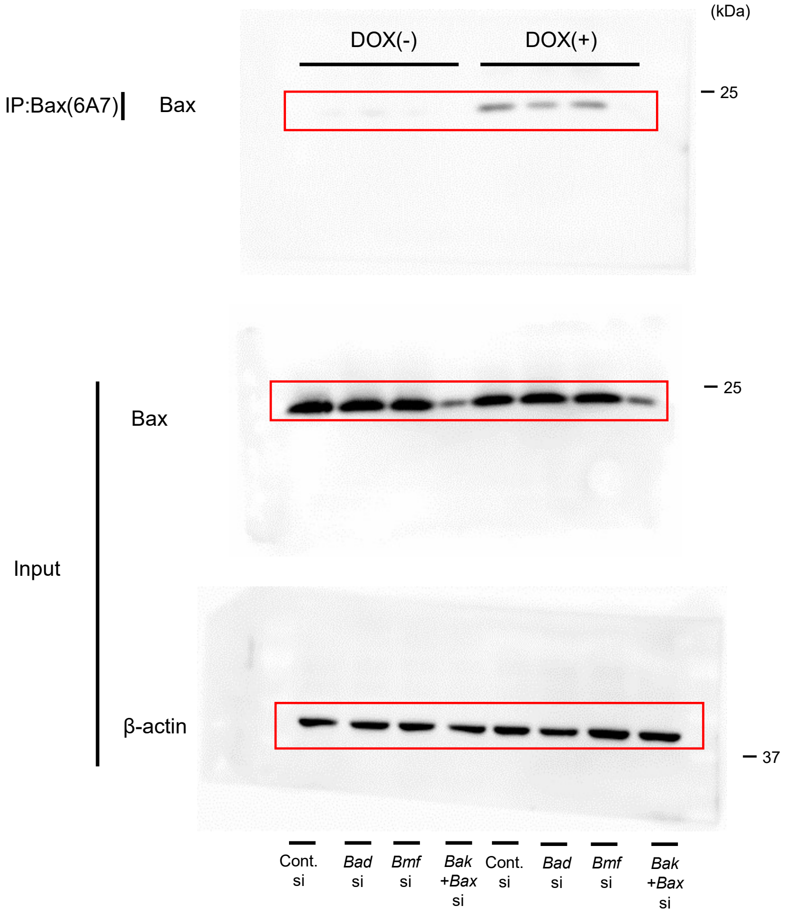

(kDa)
DOX(-)
DOX(+)
IP:Bax(6A7)
Bax
25
Input
Bax
25
β-actin
37
Cont. si
Bad si
Bmf si
Bak +Bax si
Cont. si
Bad si
Bmf si
Bak +Bax si

Fig 6H

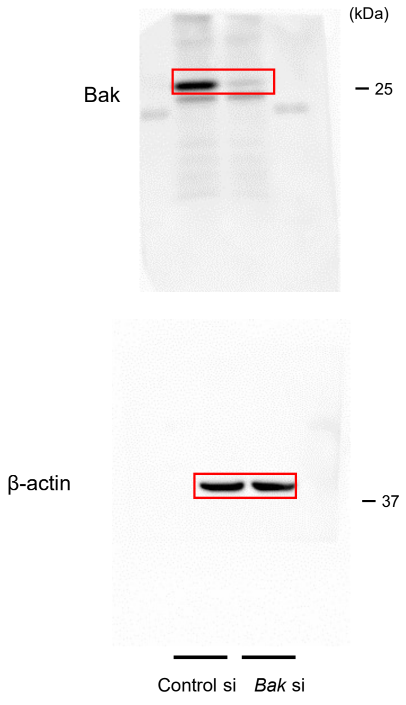
(kDa)
Bak
25
β-actin
37
Control si
Bak si

Fig 6I

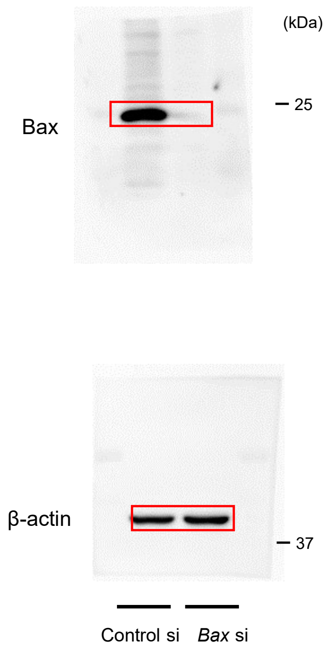
(kDa)
Bax
25
β-actin
37
Control si
Bax si

Supplementary Figure 1A

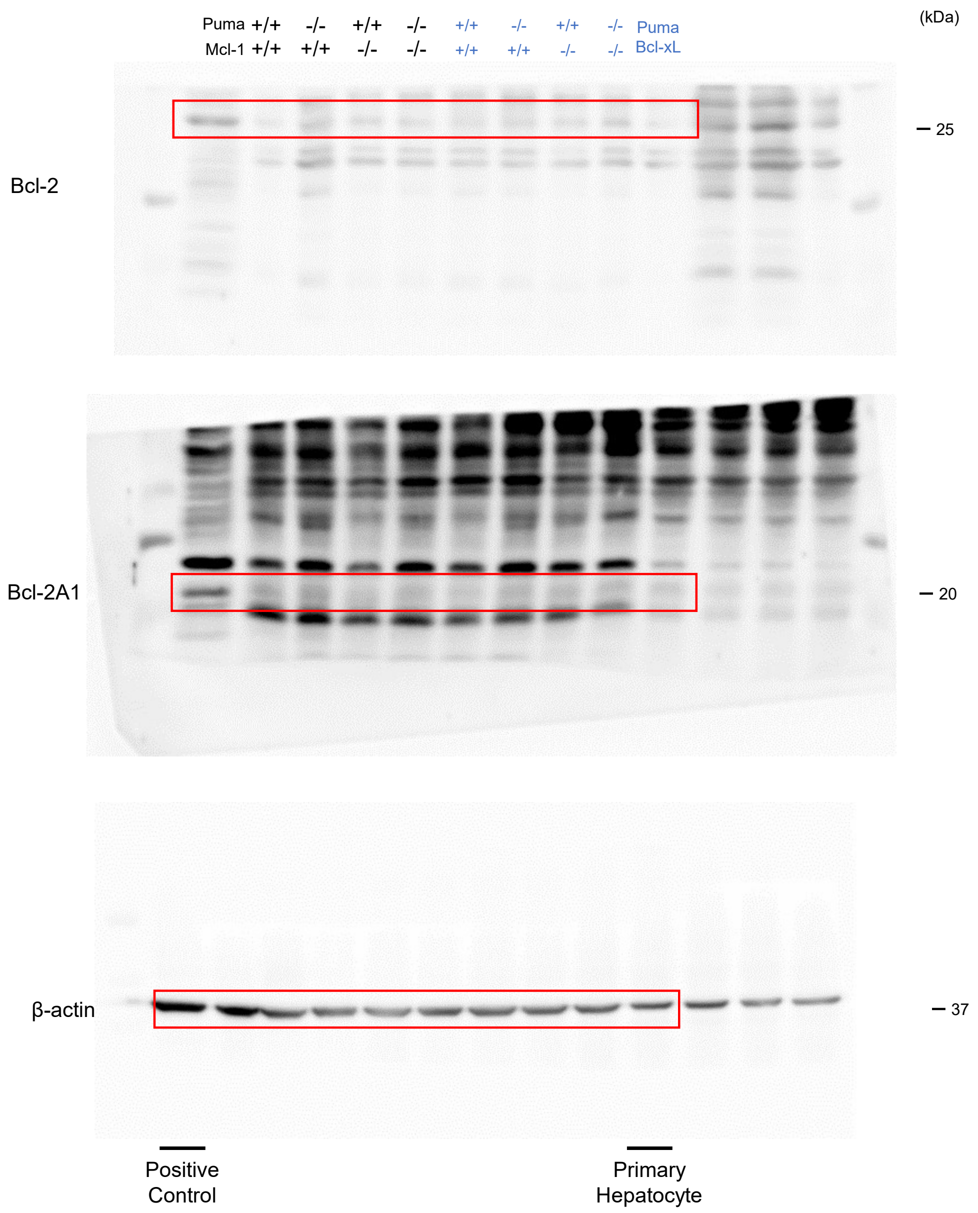

Supplementary Figure 4B

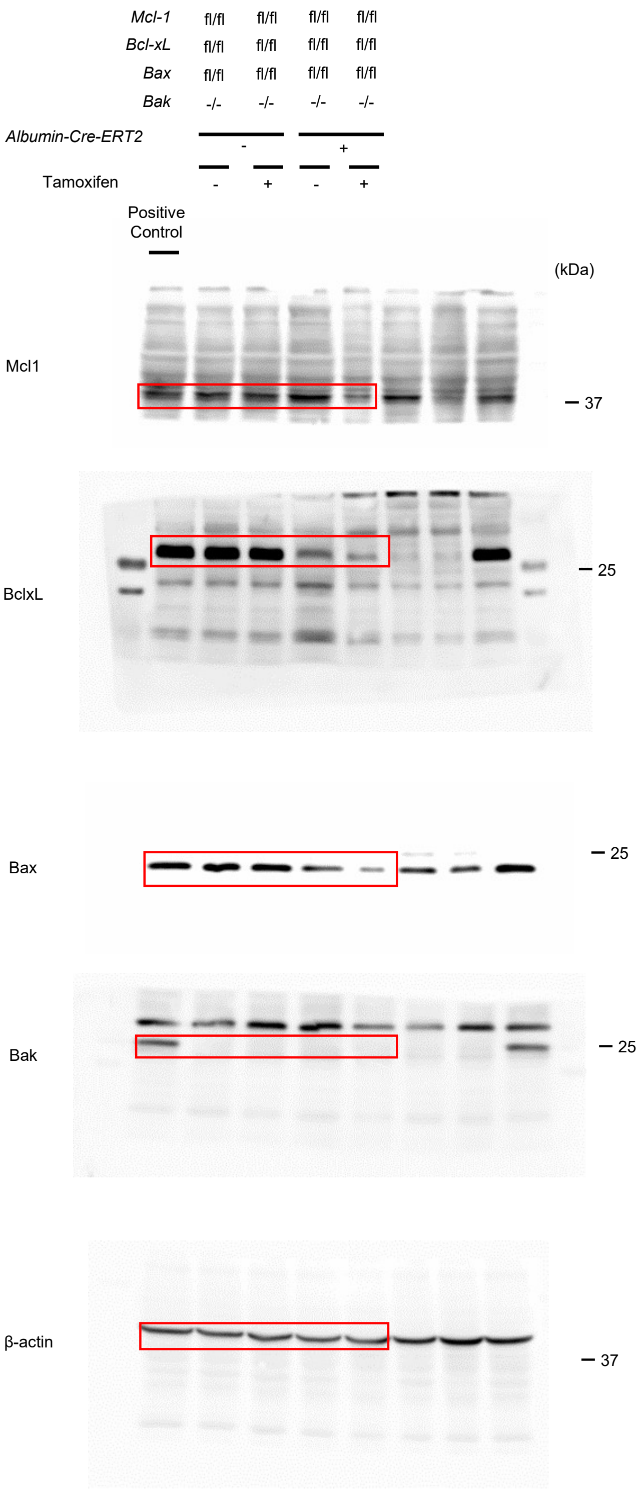

Supplementary Figure 5B

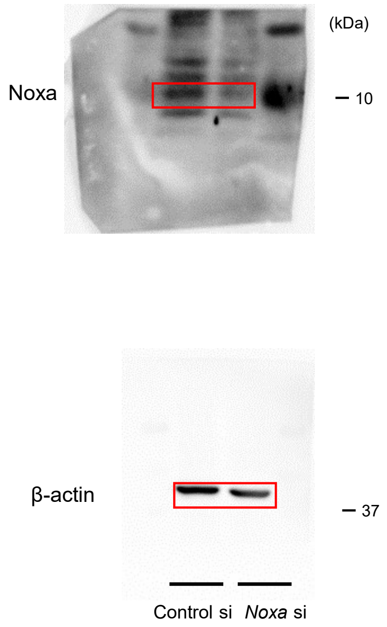

Supplementary Figure 5C

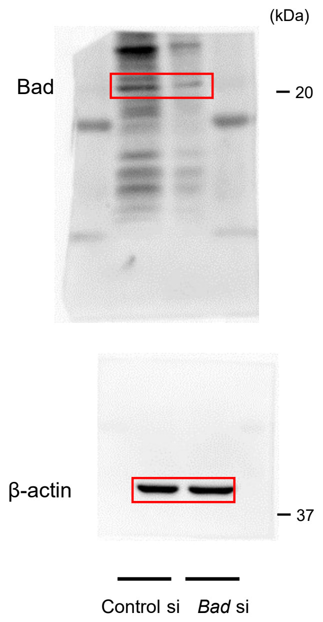

Supplementary Figure 5D

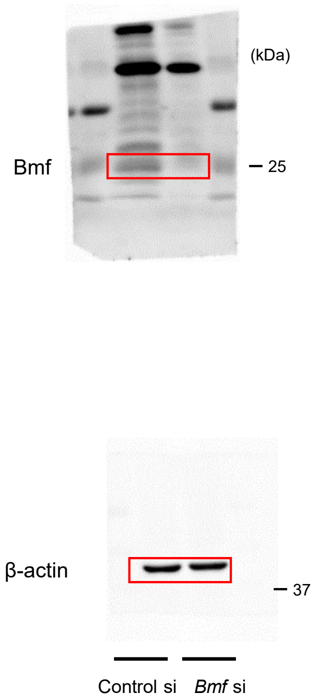

Supplementary Figure 8A

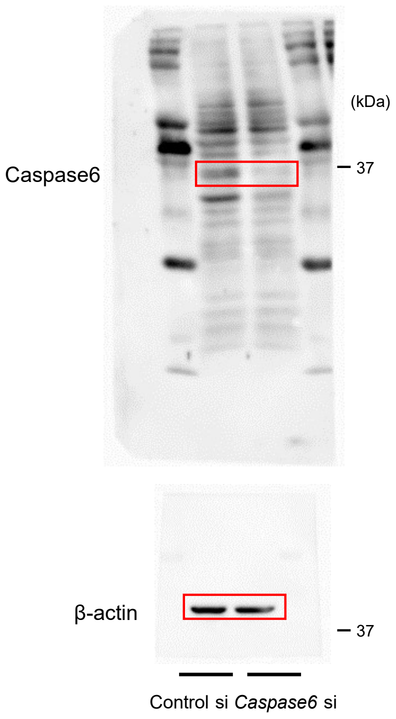

Supplement: Supplementary file 3 — Original western blots [file 41418_2025_1458_MOESM3_ESM.pdf]
